# Suite of methods for assessing inner retinal temporal dynamics across spatial and temporal scales in the living human eye

**DOI:** 10.1117/1.NPh.7.1.015013

**Published:** 2020-03-14

**Authors:** Kazuhiro Kurokawa, James A. Crowell, Furu Zhang, Donald T. Miller

**Affiliations:** Indiana University, School of Optometry, Bloomington, Indiana, United States

**Keywords:** adaptive optics, optical coherence tomography, functional imaging, retina, neurons, speckle

## Abstract

**Significance:** There are no label-free imaging descriptors related to physiological activity of inner retinal cells in the living human eye. A major reason is that inner retinal neurons are highly transparent and reflect little light, making them extremely difficult to visualize and quantify.

**Aim:** To measure physiologically-induced optical changes of inner retinal cells despite their challenging optical properties.

**Approach:** We developed an imaging method based on adaptive optics and optical coherence tomography (AO-OCT) and a suite of postprocessing algorithms, most notably a new temporal correlation method.

**Results:** We captured the temporal dynamics of entire inner retinal layers, of specific tissue types, and of individual cells across three different timescales from fast (seconds) to extremely slow (one year). Time correlation analysis revealed significant differences in time constant (up to 0.4 s) between the principal layers of the inner retina with the ganglion cell layer (GCL) being the most dynamic. At the cellular level, significant differences were found between individual GCL somas. The mean time constant of the GCL somas (0.69±0.17  s) was ∼30% smaller than that of nerve fiber bundles and inner plexiform layer synapses and processes. Across longer durations, temporal speckle contrast and time-lapse imaging revealed motion of macrophage-like cells (over minutes) and GCL neuron loss and remodeling (over one year).

**Conclusions:** Physiological activity of inner retinal cells is now measurable in the living human eye.

## 1 Introduction

The inner retina is composed primarily of ganglion cells (GCs) whose axons, somas, and dendrites locate to three distinct retinal layers: nerve fiber layer (NFL), ganglion cell layer (GCL), and inner plexiform layer (IPL), respectively. The central role of GCs in processing retinal images captured by photoreceptors^1^ has been extensively studied since the first observations of GCs by Cajal et al.^2^ However, much remains unknown about the GC neural circuitry and its vulnerability to aging and disease, in part because of our inability to observe the activity of these highly translucent cells in the living human eye.^3^^–^^8^

Recent progress in high-resolution, high-contrast imaging has overcome the translucency barrier, enabling visualization of individual retinal neurons—most notably GCs—in living human retina.^9^^–^^11^ In particular, adaptive optics optical coherence tomography (AO-OCT) allows three-dimensional (3-D) imaging of the individual cells and structures that comprise the inner retina.^10^^,^^12^^,^^13^ While successful, such imaging has not revealed the physiological activity of these cells. Here we investigate a method that does, by extending AO-OCT to detect temporal cellular changes.^14^^,^^15^ We observe temporal dynamics in the same patch of retinal tissue at dramatically different timescales, from a fraction of a second (e.g., intracellular soma dynamics) to one year (e.g., soma loss and migration). We also observe temporal dynamics on the intermediate scale of minutes, most notably the motility of macrophage-like cells—bright, irregular star-shaped cells that sparsely cover the surface of the inner limiting membrane (ILM).

We use a new correlation analysis method to characterize the fast temporal dynamics of the inner retinal layers (NFL, GCL, and IPL) and individual retinal nerve fiber bundles (RNFBs) and GCL somas. Time-lapse imaging and a temporal speckle contrast method related (but not identical) to that used in OCT angiography allow us to characterize the intermediate dynamics of macrophage-like cells and GCL somas that occur over minutes. Finally, we use pairs of images acquired one year apart to demonstrate the slow dynamics that occur over a year (neuron loss and remodeling). The ability to measure *in vivo* a wide range of fundamentally different dynamics in the same tissue using the same AO-OCT system reflects the power of the method we have developed.

## 2 Methods

Description of methods is in three sections. Section 2.1 describes the Indiana AO-OCT imaging system used in this study. Section 2.2 lays out the imaging protocol, experimental procedures, and subject information. Section 2.3 describes the postprocessing methods that we developed for visualizing and quantifying the temporal dynamics of the targeted inner retinal structures and cells. Details of our postprocessing methods are presented in Appendices A–G.

### 2.1 Indiana AO-OCT System

The Indiana AO-OCT system used in this study is described in detail elsewhere.^16^^,^^17^ Importantly, the fiber-based system operated at a center wavelength of 790 nm and bandwidth of 42 nm (superluminescent diode, SM fiber output power of 20 mW, BLMD-S-HP3, Superlum, Ireland), with a theoretical axial resolution of 4.7  μm in tissue (n=1.38) and lateral resolution of 2.4  μm (beam diameter of 6.7 mm at the eye pupil). We used the system’s two-camera mode to achieve an image acquisition speed of 500K A-scans/s (Kocaoglu et al.^17^ described the available camera modes). We focused the system on the GCL to maximize the signal strength and image sharpness of this layer. Optical power delivered to the eye was below 430  μW and more than an order of magnitude below the maximum permitted by American National Standards Institute^18^ for all our imaging protocols, as described next.

### 2.2 Imaging Protocol

Two subjects were recruited for the study (see Table 1). Neither had a history of ocular disease. The older subject was being treated for ocular hypertension (above normal ocular pressure) but was otherwise normal. All protocols adhered to the tenets of Helsinki Declaration and were approved by the Institutional Review Board of Indiana University. We obtained written informed consent after explaining the nature of the study and possible risks. Prior to the imaging session, one drop of Tropicamide 0.5% was administered to the right eye for mydriasis and partial cycloplegia. Axial eye length was measured with an IOLMaster 500 (Zeiss, Oberkochen, Germany) and used to correct for axial length differences in scaling of the retinal images following the method of Bennett et al.^19^

**Table 1:** Subject information.

Subject	Age^a^	Axial eye length (mm)	Spherical equivalent power
S1	27	24.0	0 D
S2	50	25.4	−2.5 D

a Subject age at first imaging session.

We acquired AO-OCT volume videos 12 deg temporal to the fovea. We chose this eccentricity because GCL soma sizes at the macular edge are larger and more variable,^10^^,^^20^^,^^21^ making it easier to compare cell structure and temporal dynamics. Imaging protocols for the two experiments are summarized in Table 2. Imaging protocol A was used to track fast temporal dynamics up to 2.6 Hz (Nyquist frequency of the 5.3 Hz volume acquisition rate) over a 1-deg retinal field of view and with 1-μm/A-scan lateral spacing. Imaging protocol B was used to characterize intermediate and slow temporal dynamics occurring over time durations of minutes (maximum of 16 min) and 1 year (352 days for subject S1 and 364 days for subject S2), trading off speed for a 2× increase in imaging area (1.5  deg×1.5  deg) while maintaining a sufficient volume rate to support effective 3-D image registration. All videos were time-stamped to confirm their acquisition times and to combine them for the three studies: fast, intermediate, and slow temporal dynamics.

**Table 2:** AO-OCT acquisition parameters for retinal imaging.

Imaging protocol	Image field of view (deg)	No. of A-scans per volume	Volume acquisition rate (Hz)	No. of volumes per video	Video acquisition interval (s)	No. of videos per retinal location
A	1×1	300×300	5.3	12	60±46	23
B	1.5×1.5	450×450	2.4	11 or 12	45±25	15 to 20
C^a^	0.75×0.75	150×150	20	50	48±51	30

a Protocol used for bias correction of residual eye motion (see Appendix E).

### 2.3 Postprocessing

AO-OCT volumes were registered in all three dimensions with subcellular accuracy—a process accelerated by our custom 3-D B-scan registration algorithm that registers individual fast B-scan images to a reference volume using 3-D cross correlation.^22^ Assessment of cellular structural information was enhanced by averaging registered AO-OCT volumes to reduce noise while preserving retinal content. Temporal dynamics of inner retinal layers were assessed on three different timescales: 0.38 to 2.25 s (fast), 0 to 16 min (intermediate), and 1-year interval (slow). Each timescale required different postprocessing as described below.

#### 2.3.1 Fast dynamics

**Fig. 1:**
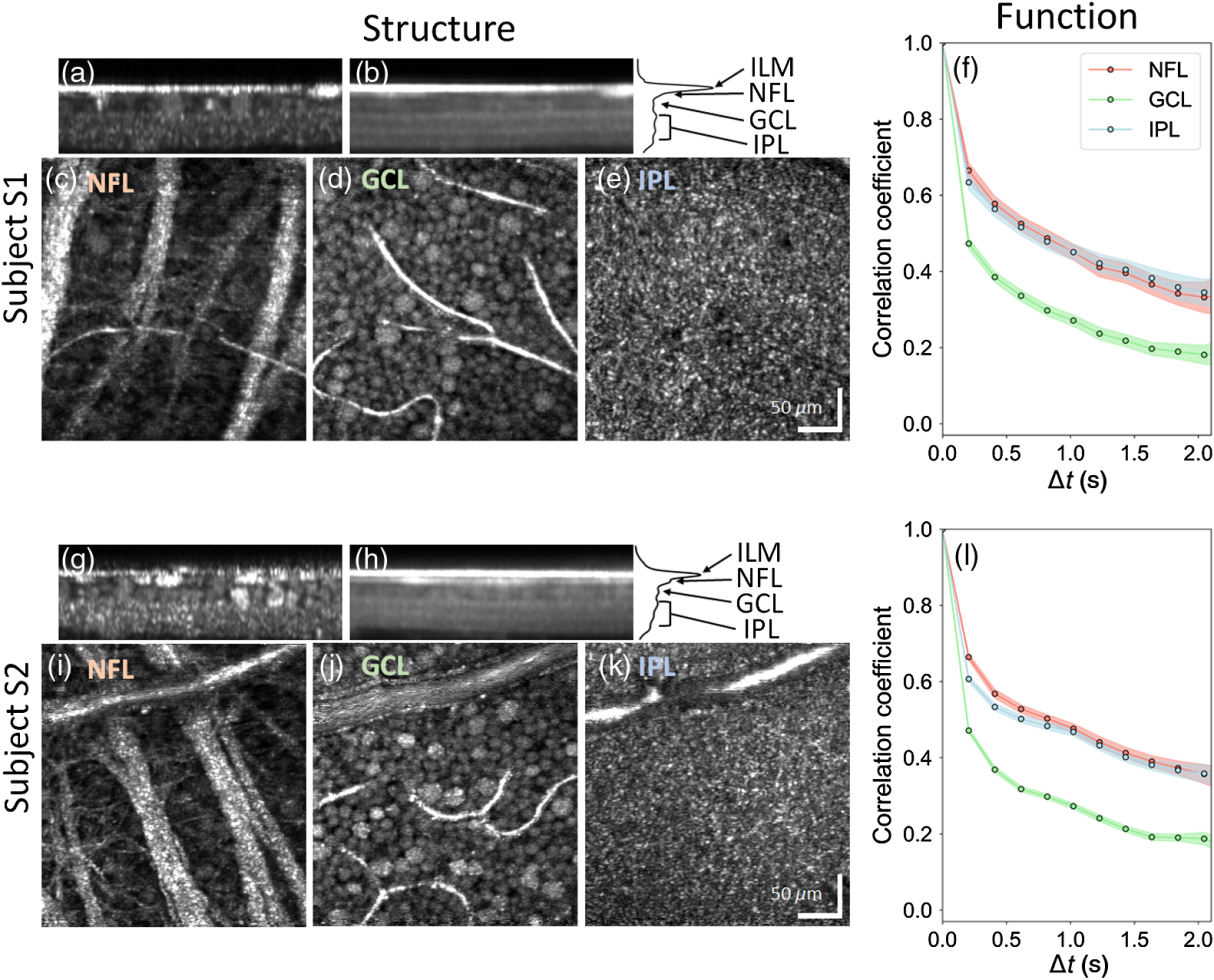
AO-OCT method reveals cellular structure and temporal dynamics of the three inner retinal layers of subjects S1 (top) and S2 (bottom). (a) and (g) Single and (b) and (h) projected B-scans show cellular and laminar reflections, respectively. Note the three hyper- and two hypo-reflective bands that compose the IPL of the inner retina, a profile we commonly observe with AO-OCT when focused at the inner retina. Labels indicate retinal depth (NFL, GCL, and IPL) at which the *en face* images in (c), (i), (d), (j), (e), and (k) were extracted. (f) and (l) Full layer (window #1) temporal correlation coefficients $\rho (\overset{\to }{r_{c}},\Delta t)$ are shown for the three retinal layers and 95% CIs (colored bands about each trace).

To characterize the fast temporal dynamics (over seconds), we developed a temporal autocorrelation method (Appendix A) that quantifies temporal change in the spatial intensity pattern within an estimation window with center pixel coordinates $\overset{\to }{r_{c}}=(X,Y,Z)$ and dimensions $\left(N_{x},N_{y},N_{z}\right)$. Given a sequence of T volume images we have a T-element time series in which each value is an $\left(N_{x}\times N_{y}\times N_{z}\right)$-element vector. We compute a correlation coefficient function $\rho (\overset{\to }{r_{c}},\Delta t)$ [Eq. (4) of Appendix A] between pairs of vectors at times t and t+Δt. This function is observed to decrease monotonically with Δt [see Figs. 1(f) and 1(l)]. The time constant τ [Eq. (5) of Appendix A] quantifies the rate of decrease. The method is considerably more complex than the conventional definition of the autocorrelation coefficient [Eq. (1) of Appendix A] in order to mitigate the effects of several key sources of error that are known to degrade the accuracy and robustness of correlation measurements: (1) biases generated by static retinal structure in the images [Eq. (2) of Appendix A], (2) bias and uncertainty generated by typical sources of measurement noise [β term in Eq. (4) of Appendix A], (3) biases generated by information loss caused by eye motion, (4) error in fitting an exponential decay to the correlation [Eq. (5) of Appendix A], and (5) bias generated by residual eye motion after image registration [Eq. (6) of Appendix A]. Based on prior studies^23^^–^^26^ we assumed optical roughness of the imaged retinal layers to be greater than the imaging wavelength, i.e., that the speckle patterns in our images were fully developed. This assumption implies that time constant τ must be independent of tissue optical roughness, a point we revisit in Sec. 4.

Correlation coefficients were computed from volume videos acquired with imaging protocol A and characterized over the range of 0.38 to 2.25 s (determined by the protocol’s 5.3 Hz volume acquisition rate and 2.25 s video length). To evaluate the trade-off between signal-to-noise (SNR) ratio and spatial resolution, we compared time constants for different retinal layers and tissue types using estimation windows of three different sizes.

The first estimation window size (window #1) covered the entire lateral extent of the volume image (typically 300×300  pixels or equivalently $300\times 300\;{\mu m}^{2}$) and was 7 pixels (6.6  μm) deep, thus including most of each retinal layer (NFL, GCL, or IPL) in depth without extending beyond it. This inclusion of hundreds of thousands of pixels yielded the most accurate time constant estimates (see Fig. 10 in Appendix A). On the other hand, each layer is composed of cellular structures of different tissue types that might have their own unique time constants, which this large window size could not differentiate.

To assess the dynamics of specific tissue types, we used a smaller estimation window size (window #2). It consisted of a 4×4×7  pixel (X,Y,Z) stack ($4\times 4\times 6.6\;\mu m^{3}$). We used this smaller window to compute a separate value of the time constant τ for each XY location at a fixed Z (depth) corresponding to the center of each retinal layer. This permitted visualization of spatial variation in temporal activity across the lateral extent of each layer. This small window size results in noisier measurements and a reduced τ because noise decorrelates. To improve the SNR ratio of our method without sacrificing tissue specificity, we averaged τ across pixels of the same nominal tissue type, which were semiautomatically determined based on differences in tissue reflectance: NFL bundles, GCL somas, GCL vasculature, and IPL synapses and processes (without vasculature).

The final estimation window size (window #3) was based on the smallest size of GCL somas in our images (7  μm) and used to more precisely evaluate their temporal dynamics. We selected pixels within a 7×7×7  pixel volume (corresponding to 7  μm in each lateral dimension and 6.6  μm in depth) centered on the soma. The volumetric center of the soma was manually identified using a customized graphic user interface display window that presents real-time *en face* (XY) and cross-sectional (XZ and YZ) slices of the AO-OCT volume image with cursor position superimposed.^15^ The 7×7×7  pixel window size captured intracellular dynamics while avoiding contributions from adjacent structures in the GCL such as glial processes, vasculature, and extracellular space.

#### 2.3.2 Intermediate dynamics

To characterize the intermediate temporal dynamics (across minutes), we constructed time-lapse image sequences of the same retinal patch from AO-OCT images acquired at different time points using imaging protocol B. A time-lapse sequence was generated for each time interval (0 to 5, 5 to 11, and 11 to 16 min for subject S1; 0 to 4, 4 to 8, 8 to 13 min for subject S2) and the motion of each pixel was quantified using temporal speckle contrast, defined as the ratio of the standard deviation (SD) of the reflectance amplitude to its mean (see Appendix B). We tested this method on GCL somas and macrophage-like cells that were observed 5  μm above the ILM.

#### 2.3.3 Slow dynamics

We characterized the slow temporal dynamics (across a year) by reimaging the same retinal patch 1 year later using imaging protocol B. Image volumes from the two time points were registered to each other using a two-step process: first, rigid displacements of the volumes were corrected using the MATLAB (The MathWorks, Natick, 2017) function *imregtform*, which iteratively optimizes image similarity using Mattes’ metric.^27^ Second, nonuniform pixel-level displacements (image warp) were corrected using the MATLAB function *imregdemons*, which iteratively optimizes local image similarities using diffeomorphic demons algorithm.^28^^,^^29^ We then analyzed the cellular-level changes between registered volumes over the intervening year. This involved visually inspecting the registered images in rapid alternation and detecting difference in cell locations.

## 3 Results

### 3.1 Fast Temporal Dynamics of Inner Retinal Layers, Isolated RNFBs, and Individual GCL Somas

Figure 1 shows the cellular structures and corresponding fast temporal dynamics (correlation coefficients) of the inner retinal layers (NFL, GCL, and IPL) of the two subjects as obtained from AO-OCT. Individual GCL somas and RNFBs are clearly delineated in the intensity images [Figs. 1(d) and 1(j)] and [Figs. 1(c) and 1(i)], respectively. Vasculature (capillaries, arterioles, and venules) are also evident in all three retinal layers. Temporal correlation coefficients $\rho (\overset{\to }{r_{c}},\Delta t)$ of the entire layers computed using window #1 (300×300×7  pixel stack) are shown in Figs. 1(f) and 1(l). NFL and IPL exhibit similar temporal dynamics, whereas GCL is clearly faster. The full-layer correlation decay time constants τ [Fig. 3(a)] confirm that GCL dynamics are ∼33% faster than those of NFL and IPL.

A two-way analysis of variance (ANOVA) tested for variations in τ with retinal layer and subject. We found a main effect of retinal layer to be significant, F(2,470)=2040, p<0.001, where 2 is the degrees of freedom of the three retinal layers and 470 is the degrees of freedom of the 476 measurements with eight total number of levels (two subjects, three layers, and three interactions). However, this main effect was qualified by a significant interaction between the retinal layer and subject, F(2,470)=12, p<0.001. There was no main effect of subjects, F(1,470)=0.1, p=0.75. Bonferroni-adjusted comparisons indicated that the time constant of GCL was significantly faster than that of NFL [p<0.001, 95% confidence interval (CI) of the difference=−0.38 to −0.32 for subject S1; p<0.001, 95% CI of the difference=−0.43 to −0.37 for subject S2] and IPL (p<0.001, 95% CI of the difference=−0.39 to −0.34 for subject S1; p<0.001, 95% CI of the difference=−0.39 to −0.33 for subject S2) of both subjects. Repeated measures on the same retinal patches gave the same results with retina layer (see Appendix G).

**Fig. 2:**
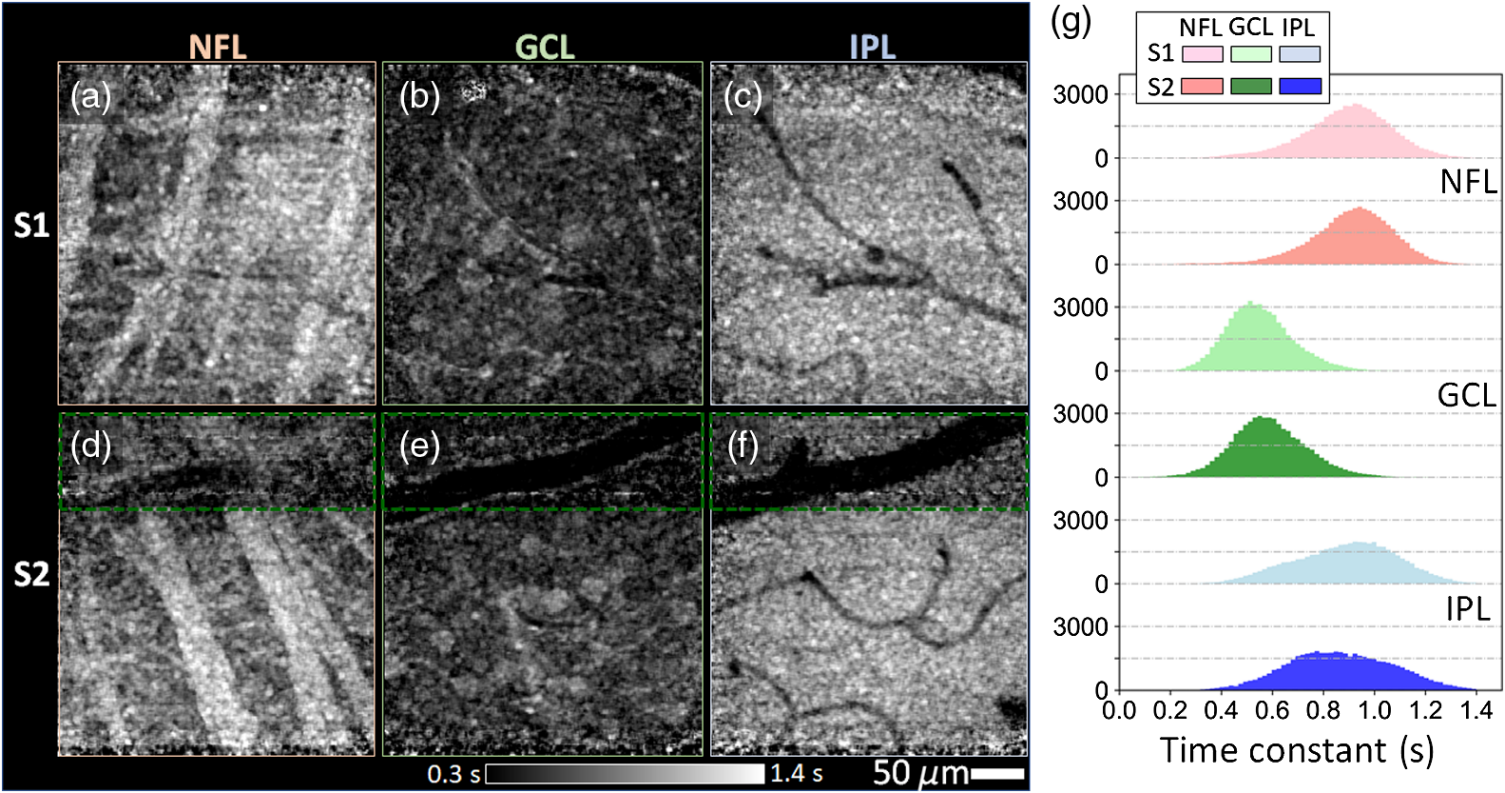
Time constants of inner retinal layers of subjects S1 (top row) and S2 (bottom row). Time constant images are shown as grayscale maps at depths of the (a) and (d) NFL, (b) and (e) GCL, and (c) and (f) IPL. Grayscale values ranged from 0.3 s (black) to 1.4 s (white). Color-coded histograms in (g) depict time constant distributions of the three layers. Note that time constants in (g) and main text exclude the region enclosed by the green dashed rectangular owing to unsatisfactory image registration caused by the large vessel.

**Fig. 3:**
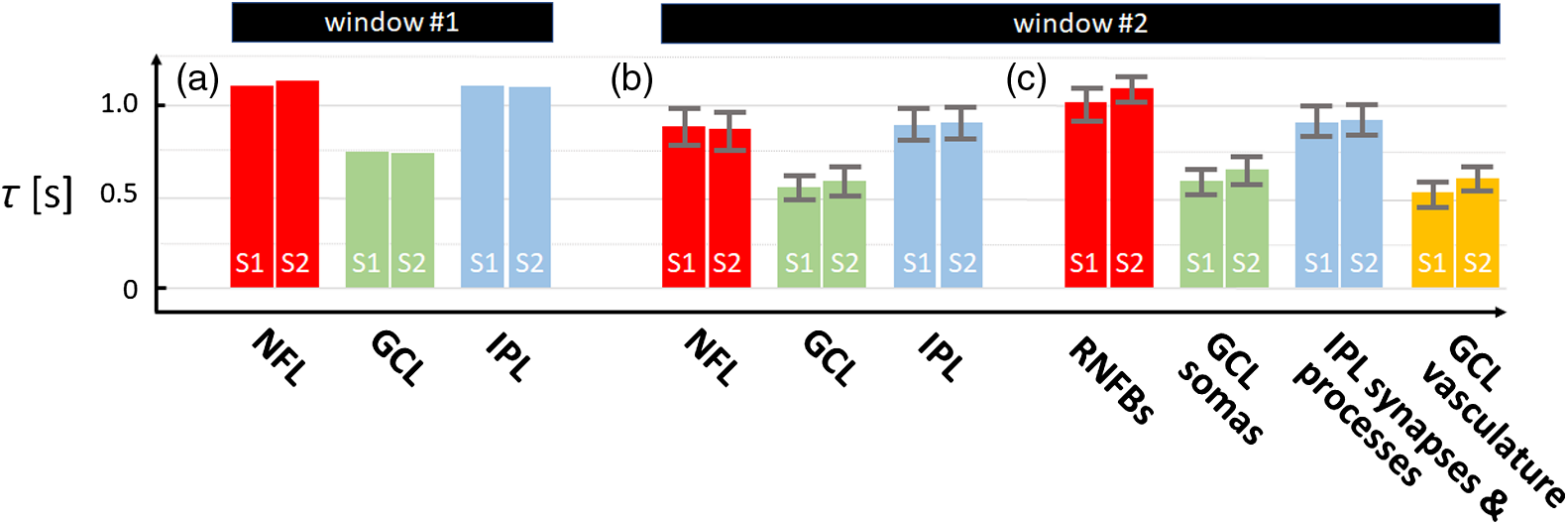
Time constants vary between retinal layer and tissue type. Time constants are shown for (a) and (b) three inner retinal layers and (c) specific tissue types within the layers using windows #1 and #2 and our AO-OCT volume data acquired with imaging protocol A. Error bars denote ±1 SD across the retinal layer or tissue type.

We assessed specific tissue-type dynamics by computing the autocorrelation using a small estimation window centered on each XY pixel location of each retinal layer (window #2—a 4×4×7  pixel stack as described in Sec. 2). The resulting spatially resolved time constants for the three layers and two subjects are shown in Figs. 2(a)–2(f) as grayscale maps with corresponding histograms in Fig. 2(g). Time constants averaged over pixels of each retinal layer are plotted in Fig. 3(b) and over specific tissue types within a layer in Fig. 3(c).

As expected, interlayer and intersubject differences show the same trends as obtained with the larger window size (window #1). However, because the smaller amount of signal pooling inherent in the smaller window #2 produced measurements with more (temporally decorrelated) noise, time constants were reduced overall by about 20%. This prevents comparison across different window sizes, but the smaller window produces spatially resolved time constant measurements and allows us to compare the dynamics of different tissue types in the same layer.

A two-way ANOVA tested for variations in τ with tissue type and subject. Both main effects were significant: tissue type {F(3,1e5)=3.4e3, p<0.001} and subject {F(1,1e5)=143, p<0.001}. However, these main effects were qualified by a significant interaction between the two, F(3,1e5)=19, p<0.001. Bonferroni-adjusted comparisons indicated that time constant of GCL vasculature was significantly faster than that of RNFBs {p<0.001, 95% CI of the difference=−0.53 to −0.46 for subject S1; p<0.001, 95% CI of the difference=−0.55 to −0.47 for subject S2}, that of IPL synapses and processes (without vasculature) {p<0.001, 95% CI of the difference=−0.41 to −0.35 for subject S1; p<0.001, 95% CI of the difference=−0.37 to −0.30 for subject S2}, and that of GCL somas {p<0.001, 95% CI of the difference=−0.10 to −0.03 for subject S1; p<0.001, 95% CI of the difference=−0.09 to −0.02 for subject S2} of both subjects.

**Fig. 4:**
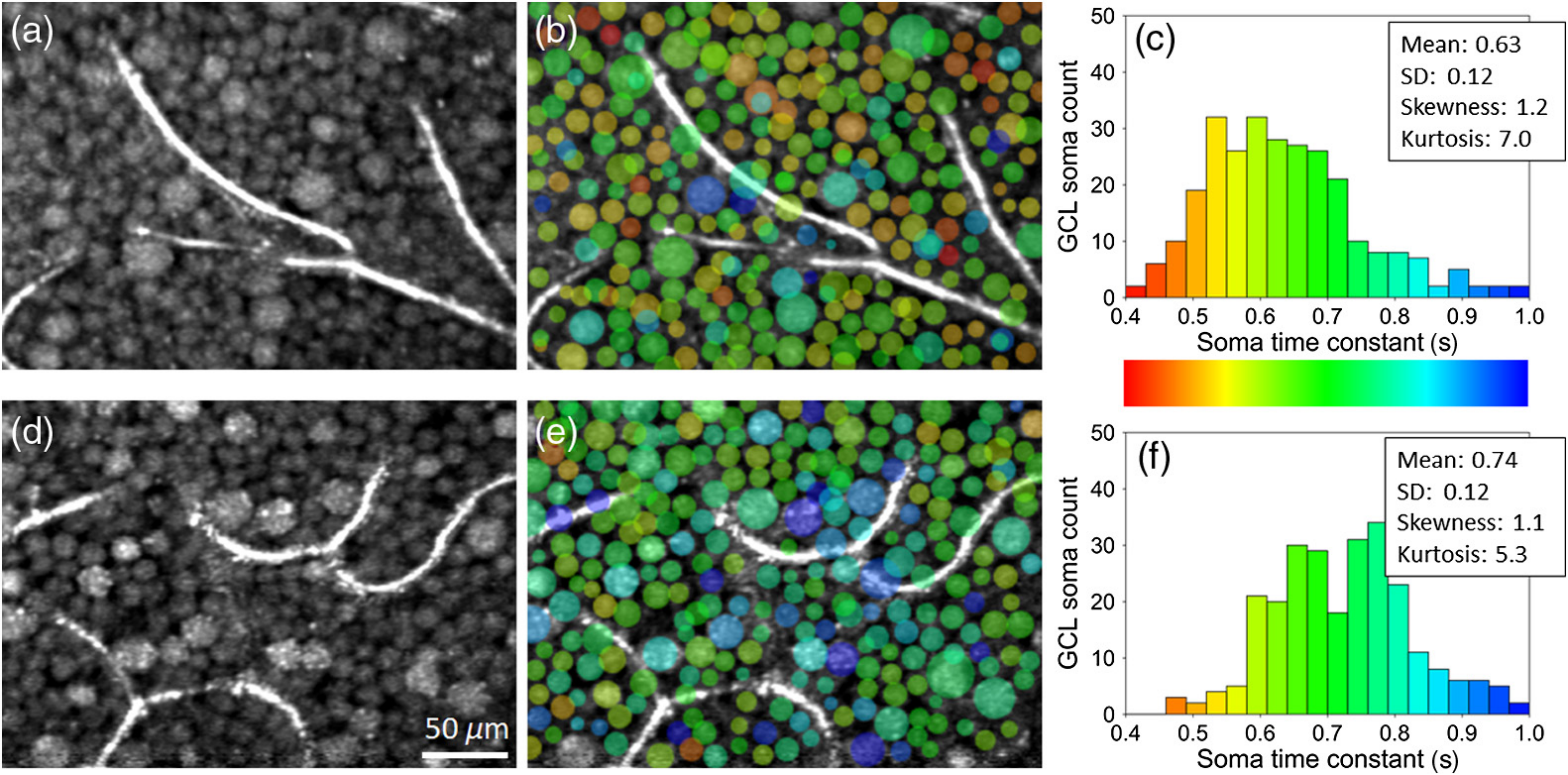
Time constants vary between GCL somas of both subjects: S1 (top) and S2 (bottom). (a) and (d) Registered and averaged reflectance amplitude images of the GCL reveal a contiguous mosaic of GCL somas disrupted only by capillaries. (b) and (e) Correlation time constants were computed for individual somas using window #3 and superimposed as semitransparent false colors as defined in (c) and (f) histograms. Note some time constant values in (b) and (e) are superimposed on capillaries and on each other as somas at these locations lie at a different depth than that of the images in (a) and (d), respectively.

We assessed individual GCL soma dynamics by computing the autocorrelation using a small estimation window centered on each soma (window #3—a 7×7×7  pixel stack as described in Sec. 2). Figure 4 shows the results, color-coded and superimposed on the corresponding reflectance amplitude image. As seen in the Figs. 4(c) and 4(f) histograms, the time constant distribution is nearly unimodal with a mean and SD of 0.63±0.12  s for subject S1 and 0.74±0.12  s for subject S2 but positively skewed (skewness=1.2 and 1.1) and leptokurtic (kurtosis=7.0 and 5.3).

### 3.2 Intermediate Temporal Dynamics of Macrophage-Like Cells at the ILM, Somas in the GCL, and Vasculature Perfusion

**Fig. 5:**
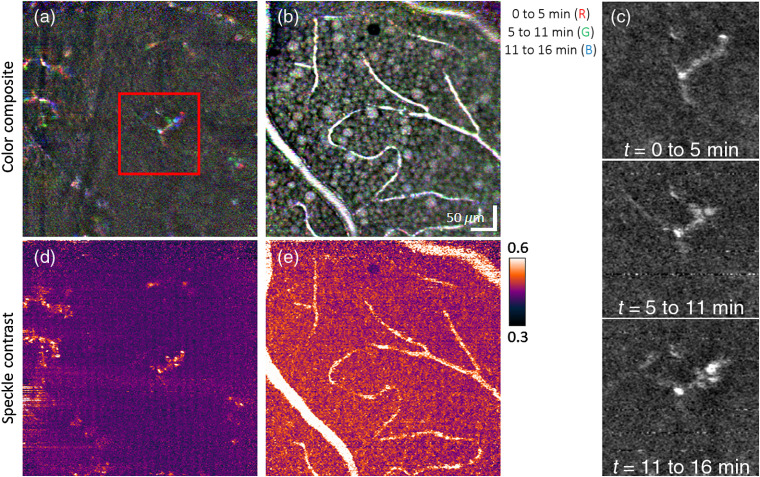
Testing for temporal dynamics of macrophage-like cells, GCL somas, and vessels over a time duration of minutes using (a)–(c) time-lapse imaging and (d) and (e) temporal speckle contrast of the same retinal patch of subject S1. Color-composite *en face* images of (a) macrophage-like cells at 5-μm vitreal of the ILM and (b) GCL somas and vessels are constructed by assigning each RGB channel to an image acquired at a different time point. Thus, colored pixels in images indicate time-lapse changes. (c) Magnified view of a macrophage-like cell in the red box of (a) and color channels (i.e., time points) displayed separately. The sequence of time-lapse images is shown in Video 1 and further substantiates the movement of macrophage-like somas and processes. Cellular dynamics and blood flow in (a) and (b) are quantified on a more local spatial scale using the temporal speckle contrast metric as shown in (d) and (e), respectively (Video 1, MPEG, 0.8 MB [URL: https://doi.org/10.1117/1.NPh.7.1.015013.1]).

**Fig. 6:**
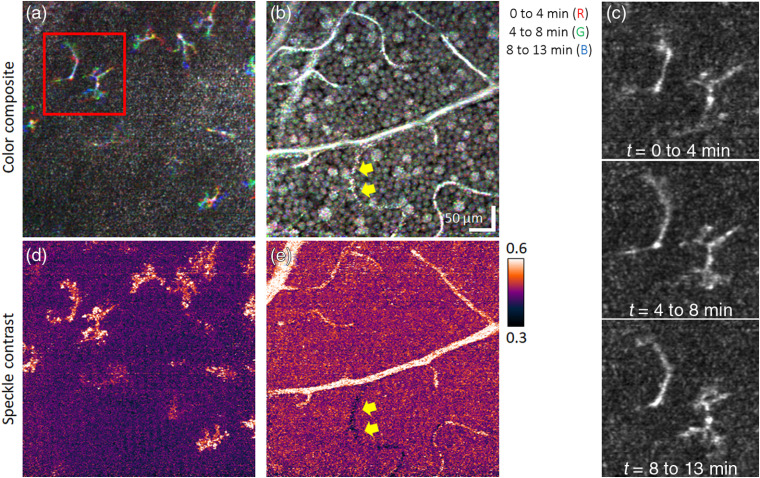
Testing for temporal dynamics of macrophage-like cells, GCL somas, and vessels over a time duration of minutes using (a)–(c) time-lapse imaging and (d) and (e) temporal speckle contrast of the same retinal patch of subject S2. Color-composite *en face* images of (a) macrophage-like cells and (b) GCL somas and vessels are constructed by assigning each RGB channel to an image acquired at a different time point. (c) Magnified view of two macrophage-like cells in the red box of (a) and color channels (i.e., time points) displayed separately. The sequence of time-lapse images is shown in Video 2 and further substantiates the movement of macrophage-like somas and processes. Yellow arrows indicate a nonperfused vessel in the GCL. Cellular dynamics and blood flow in (a) and (b) are quantified on a more local spatial scale using the temporal speckle contrast metric as shown in (d) and (e), respectively (Video 2, MPEG, 0.6 MB [URL: https://doi.org/10.1117/1.NPh.7.1.015013.2]).

Figures 5 and 6 illustrate the sensitivity of our method to temporal dynamics of macrophage-like cells, GCL somas, and vasculature perfusion over time durations of minutes. Figures 5(a), 5(b), 6(a), and 6(b) show time-lapse image triplets inserted into separate color channels of a single RGB image. Subtle movements of cells appear as color changes at the level of individual pixels (1  pixel=1  μm), whereas white/black pixels indicate absence of motion (see figure caption, for details). The sequence of time-lapse images of the macrophage-like cells, as shown in Figs. 5(c) and 6(c) and the associated videos (Videos 1 and 2), further highlights the dynamics of these cells. A more quantitative and sensitive assessment of motion in terms of speckle contrast is given in Figs. 5(d) and 6(d), revealing activity over the entire footprint of the macrophage-like soma and processes. Figures 5(e) and 6(e) depict the expected result that speckle contrast highlights those vessels that are perfused and demonstrates good mapping to the vascular structure in the intensity images in Figs. 5(b) and 6(b).

Delineation of the macrophage-like cells for subject S1 was more difficult due to uneven ILM topography, a more reflective ILM and NFL located closer to the macrophage-like cells, and reduced image quality. The stronger ILM reflection in the younger subject (S1) is consistent with that expected in younger eyes.^30^ Despite these difficulties, we observed similar motion of the macrophage-like cells in both subjects.

### 3.3 Slow Temporal Dynamics of Macrophage-Like Cells, RNFBs, and GCL Somas

**Fig. 7:**
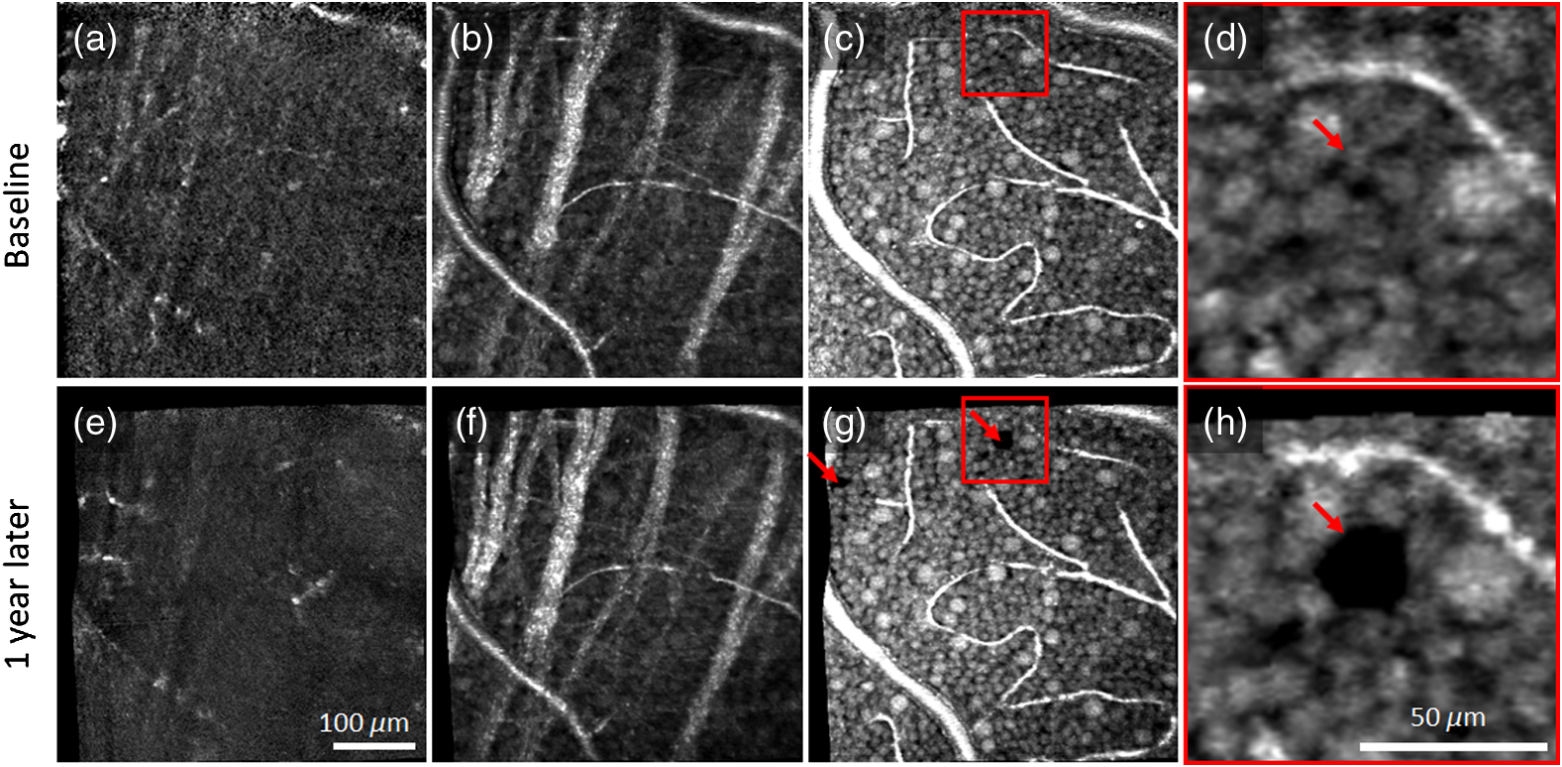
Pairs of AO-OCT images of the same patch of retina 1 year apart in subject S1 (top: baseline, bottom: 1 year later). *En face* images were extracted (a) and (e) 5-μm vitreal of the ILM where macrophage-like cells were found to reside, (b) and (f) within NFL to visualize the intricate web of RNFBs, and (c) and (g) within GCL to visualize GCL somas and vasculature. (d) and (h) Magnified view of GCL somas in the red box of (c) and (g), respectively. Video 3 highlights differences accrued over the 1-year interval by alternating between the two *en face* AO-OCT images (Video 3, MPEG, 2.1 MB [URL: https://doi.org/10.1117/1.NPh.7.1.015013.3]).

**Fig. 8:**
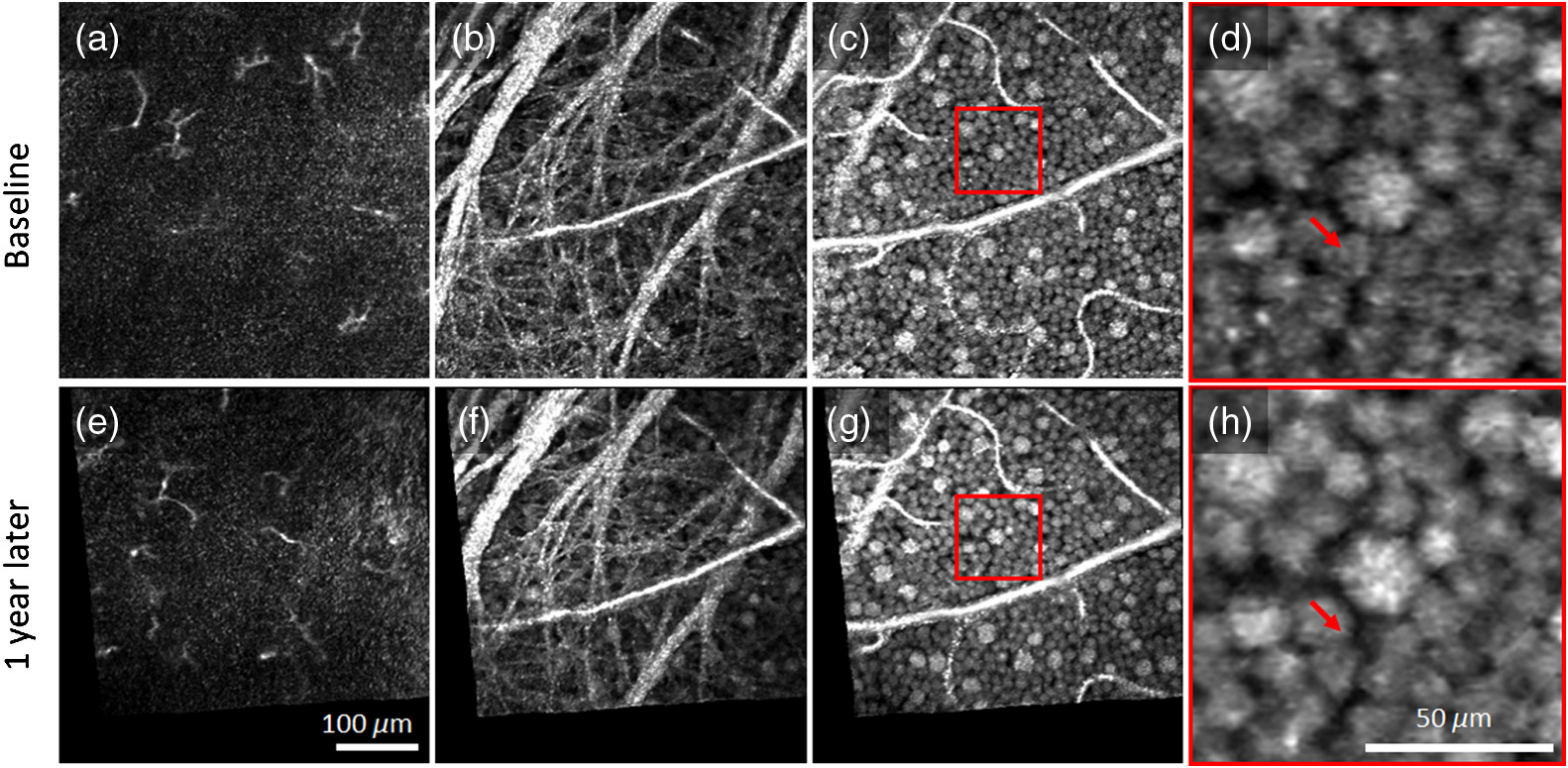
Pairs of AO-OCT images of the same patch of retina 1 year apart in subject S2 (top: baseline, bottom: 1 year later). *En face* images were extracted (a) and (e) 5-μm vitreal of the ILM where macrophage-like cells were found to reside, (b) and (f) within NFL to visualize the intricate web of RNFBs, and (c) and (g) within GCL to visualize GCL somas and vasculature. (d) and (h) Magnified view of GCL neurons in the red box of (c) and (g), respectively. The arrow in the top magnified image points to a soma that is missing 1 year later. Arrow in the bottom magnified image points to the same retinal coordinates and reveals that the surrounding somas migrated into the void left by the missing soma. No other somas were found missing. Video 4 highlights differences accrued over the 1-year interval by alternating between the two *en face* AO-OCT images (Video 4, MPEG, 2.2 MB [URL: https://doi.org/10.1117/1.NPh.7.1.015013.4]).

Figures 7 and 8 illustrate the capability of our method to assess slow temporal dynamics of macrophage-like cells and GCL somas by depicting pairs of images of the same patch of retina acquired 1 year apart. The associated videos (Videos 3 and 4) visually highlight the differences by presenting the images in rapid alternation. In contrast to the subtle motility-related changes that occur in macrophage-like cells over minutes (Figs. 5 and 6), the changes over a year are vast [Figs. 7(a), 7(e), 8(a), and 8(e)]. Macrophage-like cells appear to have been replaced between images. These cells are too active to be assessed at this timescale. In contrast, the intricate web of RNFBs in Figs. 7(b), 7(h), 8(b), and 8(h) appears stable over the whole year. Figures 7(c), 7(d), 7(g), 7(h), 8(c), 8(d), 8(g), and 8(h) demonstrate that we can also reimage the same patch of GCL somas a year later with striking one-to-one correspondence of somas and vasculature. Remarkably, there is little change in the cellular details of the GCL over this interval, indicating that the cell network is highly stable.

## 4 Discussion

### 4.1 Fast Temporal Dynamics (Seconds)

#### 4.1.1 Characterizing fast temporal dynamics

In the first part of this study, we characterized the fast temporal dynamics of the inner retina on three different spatial scales: (1) entire retinal layers, (2) structures of specific tissue types within the retinal layers, and (3) individual GCL somas. We quantified the dynamics in terms of correlation coefficients and time constants using a new correlation method (Appendix A) in conjunction with three averaging windows (denoted windows #1, #2, and #3) that defined the three spatial scales.

The largest averaging window (window #1: $300\times 300\times 6.6\;\mu m^{3}$) permitted us to separate the contributions of the individual layers (NFL, GCL, and IPL) and to achieve exceedingly small 95% CIs, as shown in Figs. 1(f) and 1(l). Across the two subjects, average time constants, τ, were 1.12±0.02  s, 0.74±0.01  s, and 1.10±0.02  s (mean±95%CI) for the NFL, GCL, and IPL, respectively [Fig. 3(a)]. As evident in these figures, the GCL was significantly more dynamic (∼33% faster) than the NFL and IPL (p<0.001).

Dynamics more specific to tissue type were obtained with smaller window #2 ($4\times 4\times 6.6\;\mu m^{3}$) with the results given in Figs. 2, 3(b), and 3(c). As expected, the fast temporal dynamics of blood flow in the vasculature and their corresponding shadows produced the smallest time constants (darkest portions of the τ images). The τ over only the segmented vasculature in GCL was 0.46±0.30  s (mean±SD), close to the shortest time duration our method can resolve (0.38 s due to the 5.3-Hz volume acquisition rate in imaging protocol A). This suggests that the actual τ for the vasculature may fall outside our measurement range. The RNFBs were the most stable, appearing white in the figure and yielding the largest time constants (1.05±0.22  s over segmented RNFBs), followed by the IPL synapses and processes (0.91±0.23  s with vasculature contributions excluded) and the GCL somas (0.62±0.20  s with vasculature contributions and intercellular space excluded).

Histograms of the three time-constant images obtained using window #2 are shown in Fig. 2(g) with modes for NFL, GCL, and IPL of 0.89±0.19, 0.55±0.13, and 0.89±0.17  s for subject S1 and 0.87±0.20, 0.59±0.15, and 0.91±0.17  s for subject S2, respectively. While some of this variance is attributable to noise, the vast majority of the pixels in the spread had an intensity >5  dB above the noise floor. Thus we interpret these as signal and attribute them to differences in temporal dynamics of the tissue. Our method is therefore sensitive enough to measure local variations in dynamics within a single retinal layer.

Finally, we assessed dynamics of individual GCL somas using window #3 (7×7×7  pixels) centered on each GCL soma. As seen in Fig. 4, the mean time constants (0.63 s for subject S1 and 0.74 s for subject S2) are consistent with those from the GCL soma measurements shown in Fig. 3(c) using window #2. The ∼3× difference between the least and most active GCL somas (τ∼0.4 to 1.0 s for subject S1 and 0.5 to 1.3 s for subject S2) is notably larger than the 95% CI of our measurement (∼0.05 for subject S1 and ∼0.07 for subject S2), indicating that we can detect differences in activity between somas.

**Fig. 9:**
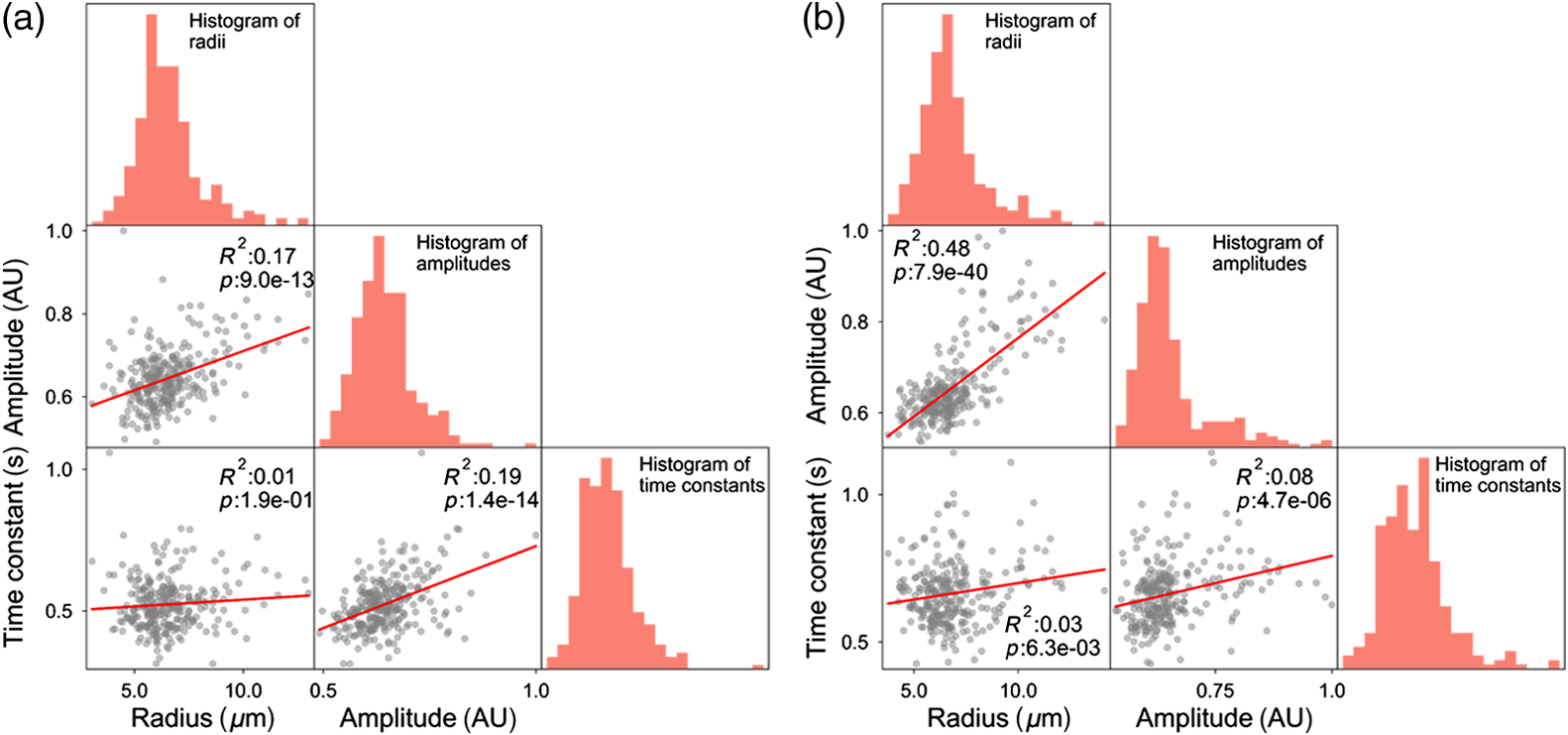
Correlation matrices of three GCL soma parameters measured in AO-OCT images: radius, reflectance amplitude, and time constant. Plots contain 279 and 268 somas for subjects (a) S1 and (b) S2, respectively. The histograms on diagonal entries are the soma radius, reflectance amplitude, and time constant distributions. Red lines are linear regression fits. $R^{2}$ and p denote the coefficient of determination and p-value.

We tested for correlations between soma activity and other soma parameters measurable in our AO-OCT volumes, namely size and reflectance. Soma size is of particular interest as it is a distinguishing feature of GC subtype (e.g., midget GC somas are smaller than parasol GC somas)^10^^,^^20^^,^^21^ at retinal eccentricities outside the fovea—such as the 12-deg eccentricity in this study. Figure 9 shows the resulting correlations. The time constant shows a weak positive correlation with soma radius, but it is significant for only one of the two subjects ($R^{2}=0.01$, p=0.19 for subject S1 and $R^{2}=0.03$, p=0.006 for subject S2). Thus, smaller somas do not particularly exhibit slower or faster dynamics compared to larger somas, at least over the temporal range that we tested (0.38 to 2.25 s).

The time constant exhibits a weak but significant correlation with soma reflectance ($R^{2}=0.19$, p<0.001 for subject S1 and $R^{2}=0.08$, p<0.001 for subject S2). Thus, somas with greater activity (smaller τ) are generally less reflective (amplitude/pixel measured), perhaps suggestive of differences in the concentrations and distributions of organelles that move about within these cells. Finally, the strongest correlation we observed was between soma radius and reflectance. This positive correlation was moderate to strong and statistically significant in both subjects (Pearson, $R^{2}=0.17$, p<0.001 for subject S1 and $R^{2}=0.48$, p<0.001 for subject S2), meaning the larger somas are generally more reflective than the smaller somas. This result is consistent with our previous finding.^10^

#### 4.1.2 Comparison to other retinal studies using speckle decorrelation

We know of no other study that has reported temporal correlation measurements of the inner retinal layers in the living human eye. It is, therefore, difficult to compare our measurements to the literature due to differences in the state of the tissue (*in vivo* versus *ex vivo*), species, retinal tissue type, and imaging and processing methods. These differences also confound attribution of our correlation measurements to subcellular activity. Nevertheless, a few comparisons to the literature are made.

The study most similar to ours, by Thouvenin et al.,^31^ measured the intracellular dynamics using correlation with full-field OCT of *in vitro* macaque and mouse retina. For macaque, temporal dynamics of 1 to 2 s or more were prevalent in RNFBs and IPL, whereas quicker dynamics of less than 1 to 2 s were dominant in GCL. They also observed that the dynamics of somas were faster than those of their surroundings. Our *in vivo* measurements in human showed the same trends with faster dynamics in the GCL than in the NFL and IPL and higher activity in GCL somas than in their surroundings. However, our measured dynamics were consistently twice as fast as theirs, perhaps due to differences in the measuring systems, experimental protocols, or state of the tissue (*in vivo* compared to *ex vivo*).

Lee et al.^32^ measured the intracellular dynamics of retinal GCs in extracted mouse retina using dynamic light scattering (DLS) OCT. They quantified the dynamics in these cells in terms of a diffusion coefficient that they reported as 1 to $4\;{\mu m}^{2}/s$. To compare, we followed Berne and Pecora^33^ by estimating an equivalent diffusion coefficient using the measured time constants of our GCL somas in Fig. 4 (see Appendix F). Based on this, the diffusion coefficient of our GCL somas was $8.0\pm 1.3\;{\mu m}^{2}/s$ ($\sigma_{i}^{2}/2$), comparable but higher than that reported by Lee et al. We also computed the diffusion coefficients of RNFBs and IPL synapses and processes using the time constants in Fig. 2 (resulting in 5.3 and $6.2\;{\mu m}^{2}/s$, respectively) but have no measurements in the literature to compare to.

Speculation surrounds the attribution of these tissue dynamics. Thouvenin et al.^31^ suggested that the dynamics they observed arise from the active transport of organelles in the cells and possible cell membrane fluctuations and surface remodeling. In particular, the cytoplasm may provide a strong dynamic, owing to the trafficking of many organelles there. Similar to Thouvenin et al., Lee et al.^32^ suggested that their dynamics attribute to the intracellular motion of relatively large organelles (0.1 to 10  μm in size, which covers the primary cell organelles). Activity-induced osmotic swelling may be another, which can occur locally within subcellular components (soma, dendrites, and axons). In photoreceptor cell outer segments, for example, growing evidence points to swelling as the dominating physical response of these cells.^34^^–^^36^ Given the general similarity of our *in vivo* measurements to the *ex vivo* ones of Thouvenin et al. and Lee et al. and the similarity of imaging methods, we expect our measurements to be sensitive to the same intracellular dynamics.

In other studies, Huang et al.^37^^,^^38^ reported much slower (τ∼34±16  s) and (τ∼59±16  s) speckle pattern dynamics in *in vitro* rat RNFBs and attributed them to axonal activity in the microtubules. This notable difference—their estimates were >15× slower than Thouvenin et al.’s and >30× slower than ours—suggests that the underlying nerve fiber bundle mechanism probed by these studies is either: (1) dramatically different in the two species; (2) slowed when removed from the eye, the extent of which depends on the method of extraction; or (3) actually two different mechanisms, perhaps because Huang et al.’s temporal sampling was 25 to 50× coarser than Thouvenin et al.’s and ours (5 and 10 s compared to 0.01 and 0.19 s). More recently, a similar difference in τ was reported *in vivo* for IPL in mouse (τ=39  s) using OCT by Zhang et al.^39^ Their temporal sampling was also considerably coarser than ours (12 s compared to 0.19 s).

Finally, two similar AO-OCT systems (including the one in this study) were used with similar scan pattern and sampling to measure organelle motility dynamics *in vivo* in human retinal pigment epithelial (RPE) cells of the outer retina.^40^ Their reported average time constant of <5  s for RPE cells is notably slower (∼5 times) than for any of the inner retinal structures we measured (∼ 1  s). Similarity of systems and protocol indicate that the speed difference cannot be attributed to our use of AO-OCT. On the other hand, we have developed a more rigorous postprocessing method to handle retinal motion and the much weaker reflections from inner retina. This difference might explain the difference in results.

#### 4.1.3 Influence of eye motion and optical roughness assumption

We mitigated key sources of error that typically affect correlation estimates. These errors included (1) biases generated by static retinal structures, (2) bias and uncertainty caused by typical sources of measurement noise, (3) biases from information loss (image gaps) due to eye motion, (4) errors in fitting an exponential decay to the correlation, and (5) bias generated by residual eye motion (after image registration). While we were careful to address the most serious errors in our imaging study, errors could have still accrued. Two of concern include the effects of residual eye motion and our assumption of fully developed image speckle (i.e., that optical roughness of the retina is greater than the imaging wavelength). We discuss these sources of error now.

We corrected for effects of eye motion at subcellular resolution using 3-D B-scan registration and then further reduced them by time averaging over different combinations of AO-OCT volumes separated by the same Δt. As stated above, we also accounted for eye-movement-related information loss within the estimation window. While we cannot be certain that these were sufficient, the correlation plots in Figs. 1(f) and 1(l) provide two lines of evidence to suggest that they were. First, the 95% CIs of the correlation coefficients (shaded colored bands) are exceedingly small (95% CI of ρ=0.02, on average). The significant differences we measured between the three layers’ correlation coefficients cannot be attributed to eye motion because they were imaged simultaneously (hence eye motion must be identical for all three layers). Second, the magnitude and pattern of eye motion is known to vary between subjects. Assuming this is true for our two subjects, a dominant effect of eye motion would have led to a difference in trends of their correlation coefficients. This was indeed observed and corrected by removing the effects of residual eye motion that manifest primarily as subpixel errors [Eq. (6) and Appendix E].

To simplify the interpretation of our results, we assumed the scattering properties of all of the layers and types of retinal tissues we examined to be “optically rough.”^23^^–^^26^ Optical roughness refers to retinal scatter within the coherence volume of the AO-OCT beam (nominally $2.4\times 2.4\times 4.7\;\mu m^{3}$) that is dominated by optical path length differences greater than the AO-OCT wavelength (790 nm), thus leading to fully developed speckle. This assumption is commonplace in characterizing tissue because of the abundance of submicron-sized organelles that densely populate cells and are known to scatter light.^25^^,^^41^^–^^43^ Optical roughness or lack thereof could significantly affect the correlation coefficient estimate. In the presence of small tissue displacements (e.g., residual eye motion), speckle for optically rough tissue (e.g., organelle-filled cells) decorrelates more rapidly than for optically smooth tissue (e.g., surface membranes), regardless of whether the displacements are corrected in postprocessing (e.g., Appendix E). Subpixel displacement of the retina could therefore affect the correlation coefficient and time constant estimates differently depending on the scattering properties of the tissue. While it is our understanding that the three retinal layers (NFL, GCL, and IPL) and tissue types [RNFBs, GCL somas, GCL vasculature, and IPL synapses and processes (without vasculature)] that we examined are approximately optically rough, we did not attempt to measure optical roughness.

### 4.2 Intermediate Temporal Dynamics (Minutes)

We examined the temporal dynamics of macrophage-like cells, GCL somas, and vasculature perfusion over a time duration of minutes using time-lapse imaging and temporal speckle contrast analysis. Direct visualization and color coding of the time-lapse videos enabled us to detect micron-scale motion of macrophage-like cell processes [Figs. 5(a), 5(c), 6(a), and 6(c)]. To the best of our knowledge, these are the first observations of macrophage-like cell dynamics in the living human retina. The cells’ few stout processes and their apparent random distribution in a narrow region just anterior to the ILM suggest that they might be hyalocytes, a subtype of macrophage-like cell that typically resides in the cortical vitreous, either adjacent to or abutting to the retinal surface.^44^^–^^47^ Hyalocytes share a common origin^44^^,^^48^ and similar dynamics with microglial cells.^46^^,^^47^^,^^49^ Both are scavenging cells that continuously probe their local microenvironment. The cellular motion we observed was consistent with that reported for fluorescence-labeled microglia in *ex-vivo*^50^ and *in-vivo*^51^ experiments using mice, and so in our earlier report^10^ we interpreted the cells to be microglial or possibly astrocytes. However, microglial cells primarily populate the GCL and the inner and outer plexiform layers, so interpretation as hyalocytes is more plausible. Whatever their type, our methods have the sensitivity and temporal resolution to detect and track these cells *in vivo* as they scavenge about the retinal surface.

Our time-lapse results of the GCL [Figs. 5(b) and 6(b)] reveal—as expected—a highly stable GCL soma mosaic and vasculature over the time duration of minutes. These images show clear demarcation of the vasculature network, including small capillaries, but fail to differentiate perfused from nonperfused vessels. However, the temporal speckle contrast metric reveals subtler dynamics in the time-lapse videos [Figs. 5(e) and 6(e)]. The largest values are detected in the vasculature due to blood flow (color-coded as white), but we also observe an elevation or bias that permeates the entire GCL (GCL somas and extracellular space), color-coded as orangish red in the figures. This bias is greater than the system sensitivity as measured in the vitreous at the corresponding macrophage-like cell layer [Figs. 5(d) and 6(d)], color-coded as dark violet. The same layer demonstrates motion of individual macrophage-like cells, revealing activity over the entire footprint of the cell’s soma and processes and is consistent with our direct visual inspection of the time-lapse videos.

Interestingly, this speckle contrast metric permitted us to identify one nonperfused capillary. This capillary—located at the bottom of the speckle contrast image in Fig. 6(e) (yellow arrows)—is identifiable by its strikingly dark appearance compared to the other vessels. Dysfunction of this vessel is not evident in the corresponding color-coded time-lapse image [Fig. 6(b)].

### 4.3 Slow Temporal Dynamics (1 Year Interval)

In pairs of images acquired 1 year apart and inspected visually in rapid alternation, macrophage-like cells caused the most obvious image changes, with the same cells likely not present in both images (Figs. 7 and 8). This degree of activity is consistent with the scavenger role of macrophages, which are known to migrate across and through the retina. As they have been implicated in the pathogenesis of numerous retinal diseases, their numbers are believed to fluctuate as a function of retinal health. We can now measure their numbers and track them longitudinally.

The cells that compose the GCL appear highly stable over the 1-year interval except for abrupt changes associated with GCL soma loss and remodeling. For the GCL patches shown in Figs. 7(c) and 7(g) of subject S1 and in Figs. 8(c) and 8(g) of subject S2, we identified 831 and 589 somas, respectively, that were present at both times. We also identified one soma in subject S2 that was present in the first image but not the second. This missing soma is more salient in magnified view in Figs. 8(d) and 8(h), which also reveal migration of neighboring somas into the void created by the vanished soma that thus represents a form of retinal remodeling at the cellular level. Loss of 1 out of 590 GCL neurons is consistent with the histological reports of aging-related loss (0.19 to 0.72%/year^52^^–^^56^) and within the range of loss rates that we have reported in an ongoing AO-OCT study of five different retinal locations in each of four normal subjects.^57^ The ability of our method to detect the loss of a single GCL soma demonstrates potential for extremely early detection of onset of GC-affecting diseases such as glaucoma.

Interestingly, we observed an unidentified dark globoid in subject S1 [Figs. 7(d) and 7(h)] that formed over the 1-year interval and appeared to have displaced adjacent GCL somas. The 24-μm diameter feature generates bright reflections at its top and bottom boundaries. Its size and reduced internal reflectance are consistent with a displaced soma from the inner nuclear layer, e.g., a Müller cell (which are known to sometimes displace to the GCL^58^^,^^59^). However, unlike neighboring GCL somas, no internal structure is evident, so it may instead be a fluid-filled vacuole (microcyst). The nearest vessel that could supply fluid is ∼20  μm away. A second smaller globoid is evident at the far left of the image (second red arrow). In subsequent imaging sessions of this same retinal patch, the larger globoid disappeared over 12 months, whereas the smaller one increased in size. We have since identified similar globoids in the GCL of other subjects.

## 5 Conclusion

We have developed a noninvasive method based on AO-OCT and a suite of novel postprocessing methods that measures both structural and physiological activities in retinal tissue down to the level of individual cells in the living human eye. The method was successfully applied to quantify the temporal dynamics of entire inner retinal layers, of specific tissue types, and of individual cells across three different timescales. Detecting physiological dynamics in this way offers the exciting possibility of longitudinally tracking very early cellular changes associated with disease onsets that cannot currently be detected clinically. This new capability also advances the prospects for noninvasively mapping functional aspects of neural circuitry in the living human retina.

## Appendix A: Temporal Autocorrelation Method

Temporal autocorrelation is already used in Doppler OCT and OCT angiography. These methods are designed to detect rapid changes that occur on the scale of microseconds to milliseconds,^60^^,^^61^ a range fast enough for measuring blood flow (e.g., velocity ranging from ∼1 to 35  mm/s^62^^–^^64^). For our application of measuring subcellular organelle motility, the changes we sought to detect are entirely diffusive (random motion; no flow) and occur over a much longer time period (couple of seconds). These translate into a variance rate from 5 to $29\;\mu m^{2}/s$ for our AO-OCT method (see Appendix F). Such slow dynamics (well below that detected by Doppler OCT and OCT angiography) exposes our method to eye motion artifacts and therefore requires careful attention to minimize these artifacts.

Starting with these initial requirements, we developed a correlation method using the conventional definition of the autocorrelation coefficient:

$$\rho (\overset{\to }{r},\Delta t)=\frac{{\left\langle A(\overset{\to }{r},t)A(\overset{\to }{r},t+\Delta t)\right\rangle }_{T}}{{\left\langle A^{2}(\overset{\to }{r},t)\right\rangle }_{T}},$$ (1)

where $A(\overset{\to }{r},t)$ and $A(\overset{\to }{r},t+\Delta t)$ are the measured reflectance amplitudes at pixel location $\overset{\to }{r}$ in the AO-OCT volume image and acquired at times t and t+Δt, respectively, and ${\left\langle \right\rangle }_{T}$ denotes the temporal averaging. Our method was designed to mitigate key sources of error that are known to affect correlation estimates. These errors include (1) biases generated by static retinal structure, (2) bias and uncertainty generated by typical sources of measurement noise, (3) biases generated by information loss caused by eye motion, (4) error in fitting an exponential decay to the correlation, and (5) bias generated by residual eye motion (after image registration). Established methods to handle most of these errors can be found in DLS theory^33^ (static structure; nonexponential decay), averaging and Rician- or Rayleigh-noise corrected correlation estimation (measurement noise^65^^–^^69^), and masked correlation^70^ (information loss). We customized each of these to our application (imaging in the living human retina) and then combined them to create a unique sequence that mitigates our key sources of error. New are our methods to handle eye motion, including information loss and residual subpixel-level motion. These were implemented as follows.

First, to avoid biases generated by static retinal structure, time-invariant contributions in the AO-OCT volumes were removed by subtracting the time average of each pixel:

$$A^{\prime }(\overset{\to }{r},t)=A(\overset{\to }{r},t)-{\left\langle A(\overset{\to }{r},t)\right\rangle }_{T},$$ (2)

where ${\left\langle A(\overset{\to }{r},t)\right\rangle }_{T}$ is the temporal average over time duration T of the measured reflectance amplitude at each pixel location and thus contains only static structural information. By empirical assessment, we set T to be 15 min as this duration is significantly longer than the fluctuation period τ, the time constant of the tissue defined by Eq. (5) below and found in this study to be ∼1  s. τ≪T assured structural correlation bias was removed from time constant measurements (see Appendix C, for the effect of T).

Second, we reduced the uncertainty and bias caused by noise. For the former, we performed both temporal and spatial averaging. Temporal averaging was realized by averaging ρ over all possible volume combinations of Δt within a given volume video and across all volume videos acquired of the same retinal patch. For our study, the temporal sample size ranged from 23 to 253, depending on Δt. Further improvement was gained by computing the mean correlation between $A^{\prime }(\vec{r},t)$ and $A^{\prime }(\overset{\to }{r},t+\Delta t)$ across a 3-D spatial estimation window w centered on pixel location $\overset{\to }{r}$. This entailed first removing spatially invariant contributions by subtracting the local spatial average: $A^{\prime \prime }(\overset{\to }{r},t)=A^{\prime }(\overset{\to }{r},t)-\langle A^{\prime }(\overset{\to }{r},t)\rangle_{\overset{\to }{r}\in w}$, where $\langle \rangle_{\overset{\to }{r}\in w}$ denotes the spatial averaging over w. With these changes, Eq. (1) becomes

$$\rho (\overset{\to }{r},\Delta t)={\left\langle \frac{\langle A^{\prime }(\overset{\to }{r},t)\cdot A^{\prime }(\overset{\to }{r},t+\Delta t)\rangle_{\overset{\to }{r}\in w}}{\sqrt{\langle {\left[A^{\prime }(\overset{\to }{r},t)\right]}^{2}\rangle_{\overset{\to }{r}\in w}{\left\langle [A^{\prime }(\overset{\to }{r},t+\Delta t)\right]}^{2}\rangle_{\overset{\to }{r}\in w}}}\right\rangle }_{T}.$$ (3)

In our study, we used w sizes of 4×4×7, 7×7×7, and 300×300×7  pixels, denoted as window #2, #3, and #1 in the main text. Because the average speckle size in our AO-OCT images was approximately equal to the nominal optical resolution of the AO-OCT (2.4×2.4×4.7  μm in retinal tissue) and image sampling was ∼1  μm/pixel in all directions, the effective number of independent samples occupying each window size was roughly 4, 13, and 23,000, respectively.

**Fig. 10:**
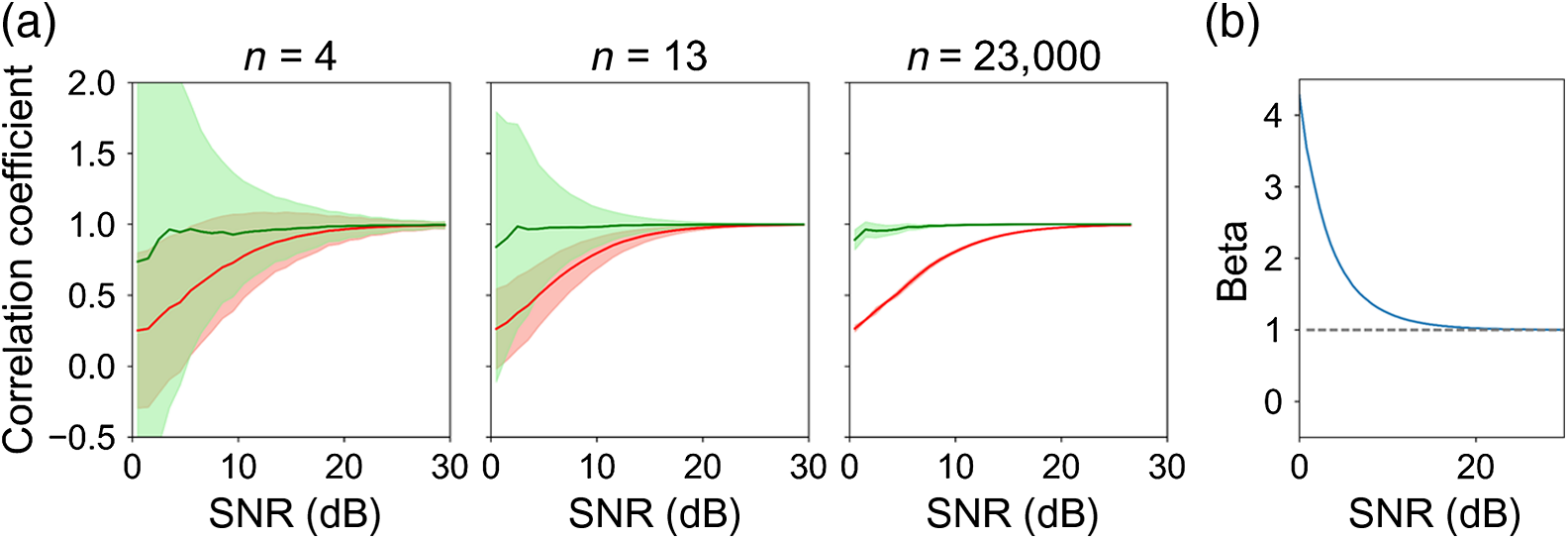
Effectiveness of β in Eq. (4) to correct Rayleigh distributed noise as a function of SNR and spatial averaging. (a) The Monte-Carlo simulation shows the predicted correlation coefficient for different numbers of independent samples (n=4, 13, and 23,000) that correspond to the three different window sizes used in our AO-OCT study. Red solid trace and shaded error bar denote mean and SD of correlation coefficients without correction. Green solid trace and shaded error bar denote mean and SD of correlation coefficients with bias correction. For this simulation, the true correlation coefficient was set to 1 for all values of Δt. (b) The estimated β used in this study was set to the inverse of the average uncorrected correlation coefficient (red solid trace) for the n=23,000 case.

Noise can also create a bias in the correlation estimate that artificially attenuates ρ ^71^^,^^72^ and reduces the corresponding estimated time constant, τ (defined below). This can be particularly problematic for additive pixel-correlated noise such as the photon noise, read noise, and relative intensity noise that are all present in our AO-OCT volume images. To correct this noise bias, we modified Eq. (3) as

$$\rho (\overset{\to }{r},\Delta t)={\left\langle \frac{{\left\langle A^{\prime \prime }(\overset{\to }{r},t)\cdot A^{\prime \prime }(\overset{\to }{r},t+\Delta t)\right\rangle }_{\overset{\to }{r}\in w}}{\sqrt{{\left\langle {\left[A^{\prime \prime }(\overset{\to }{r},t)\right]}^{2}\right\rangle }_{\overset{\to }{r}\in w}\langle {\left[A^{\prime \prime }(\overset{\to }{r},t+\Delta t)\right]}^{2}\rangle_{\overset{\to }{r}\in w}}}\cdot \beta [\mathrm{SNR}(\overset{\to }{r},t)]\right\rangle }_{T},$$ (4)

where the new variable $\beta [\mathrm{SNR}(\overset{\to }{r},t)]$ is a multiplicative weighting factor of signal strength that compensates for the bias in ρ and τ due to additive noise. Here $\mathrm{SNR}(\overset{\to }{r},t)$ is the measured signal-to-noise ratio defined as $\{{\left\langle {\left[A^{\prime \prime }(\overset{\to }{r},t)\right]}^{2}\right\rangle }_{\overset{\to }{r}\in w}-N\}/N$, where the pixel-correlated noise N is determined from AO-OCT pixels in the vitreous whose values are dominated by noise. We estimated $\beta [\mathrm{SNR}(\overset{\to }{r},t)]$ using a Monte Carlo simulation assuming that the signal is free of specularity (no specular reflection) and that both signal and noise are zero-mean complex Gaussian variables whose amplitudes thus follow a Rician (or Rayleigh) distribution.^26^^,^^41^^–^^43^^,^^73^^,^^74^ More specifically, we first generated two correlated complex vectors ${\overset{\to }{s}}_{1}$ and ${\overset{\to }{s}}_{2}$, each of length equal to the number of speckles within an averaging window of set size. We controlled the correlation between the two vectors using Cholesky decomposition and also controlled the signal strength by multiplying both vectors by an adjustable constant. We also generated two uncorrelated noise vectors ${\overset{\to }{n}}_{1}$ and ${\overset{\to }{n}}_{2}$. These were added in the complex domain to the two signal vectors, and their absolute values (i.e., $\left|{\overset{\to }{s}}_{1}+{\overset{\to }{n}}_{1}\right|$ and $\left|{\overset{\to }{s}}_{2}+{\overset{\to }{n}}_{2}\right|$) were used to compute correlation coefficients. To model temporal averaging, we generated 1000 pairs of vectors and then computed the mean and SD of their correlation coefficients. While Fig. 10 shows only the case for the desired correlation equal to 1 for simplicity, we confirmed that this bias correction is valid for any desired correlation. Similar approaches have been proposed, for example, for estimating and correcting systematic error in diffusion tensor magnetic resonance imaging,^65^ polarization-sensitive OCT,^66^^,^^67^ and OCT angiography.^68^^,^^69^ Here our method was designed for correcting errors in computing correlation coefficients based on AO-OCT amplitude signals.

Figure 10(a) shows the simulated correlation coefficient $\rho (\overset{\to }{r},\Delta t)$ both without (red trace) and with (green trace) β correction for different levels of spatial averaging (n=4, 13, and 23,000). The true value of $\rho (\overset{\to }{r},\Delta t)$ was set to 1. This figure reveals two key findings. First, the notable differences between the red and green traces substantiate the importance of correcting for Rayleigh distributed noise bias, especially at low SNR. Second, the reduction in the widths of the green and red shaded regions as n increases indicate that more precise estimates result from increased spatial averaging. Further improvement is possible by also performing temporal averaging, which reduces standard error of the mean (SEM) of the correlation coefficients. We employed temporal averaging in processing our data but not in the Monte Carlo simulation. Figure 10(b) shows the estimate of β we used in our study, obtained with the largest window size (n=23,000).

Note that the use of fixed windows for spatial averaging exposes our correlation estimates to eye motion biases. An eye movement can cause the imaging beam to skip over a region of retina, yielding a gap in the volume image within a given estimation window. This corresponds to error source #3: biases generated by information loss caused by eye motion. To avoid this window bias, we automatically masked those unimaged pixels in the averaging window.

Next, we estimated the time constant, τ, by summing the time-averaged correlation coefficients across Δt:

$$\tau (\overset{\to }{r})=\overset{2.25s}{\underset{\Delta t=0s}{\sum }}\rho (\overset{\to }{r},\Delta t).$$ (5)

This general expression for τ avoids the assumption of an exponential decay (error source #4), which our data did not follow. Note that the discrete integral in Eq. (5) theoretically underestimates τ because it is bounded at 2.25 s, the maximum time duration of our AO-OCT videos. While τ is sensitive to the maximum time duration (quantified in Appendix D), we used 2.25 s as it captures the period of most rapid change in the NFL, GCL, and IPL and also avoids unwanted disturbances, such as eye blinks and tear film breakup.

Finally, our image registration algorithm corrects motion artifacts as small as a single image pixel, but this leaves subpixel-level artifacts that can bias the correlation coefficient and time constant, which is also known as “spatial decorrelation noise” in Doppler OCT and OCT angiography.^74^^–^^76^ Because speckle noise dominates our AO-OCT images, we described this decorrelation bias as^74^^–^^76^

$$\sigma_{\rho }^{2}=\exp[-\underset{i=x,y,z}{\sum }{\left(\frac{\varepsilon_{i}}{w_{i}}\right)}^{2}],$$ (6)

where $w_{i}$ is the speckle size of our AO-OCT system (2.4, 2.4, and 4.7  μm for i=x,y,z in retinal tissue) and $\varepsilon_{i}^{2}$ is the residual displacement errors [variance ($\mu m^{2}$)] in each axis direction (i=x,y,z). We estimated $\varepsilon_{i}^{2}$ from repeated measures of the same retinal patch of each subject (see Appendix E). Using Eq. (6), we obtained a multiplicative bias correction factor for the residual eye motion ($1/\sigma_{\rho }^{2}$) and then applied the factor to both $\rho (\overset{\to }{r},\Delta t)$ and τ (see Appendix E).

We computed the SEM to assess confidence limits of our ρ and τ estimates. SEM of τ was determined by summing the squares of the ρ SEMs and taking the square root.

## Appendix B: Temporal Speckle Contrast

We used temporal speckle contrast, an established motion metric based on time-varying speckle,^73^^,^^77^ to quantify intermediate AO-OCT image temporal dynamics (across minutes). Temporal speckle contrast is defined as the ratio of the SD of the reflectance amplitude to its mean:

$$C(\overset{\to }{r})=\frac{\sqrt{{\left\langle {\left[A(\overset{\to }{r},t)-\langle A(\overset{\to }{r},t)\rangle_{T}\right]}^{2}\right\rangle }_{T}}}{{\left\langle A(\overset{\to }{r},t)\right\rangle }_{T}},$$ (7)

where all variables are defined as in Eq. (1) in Appendix A. Temporal speckle contrast defined in this way is independent of the average reflectance amplitude and reaches a maximum theoretical value of 0.52 for fully developed speckle.^26^^,^^41^^–^^43^ This maximum value for the reflectance amplitude is equivalent to 1 for the corresponding reflectance intensity, a difference attributable to their different probability density functions (Rayleigh versus exponential).^25^^,^^41^^,^^42^^,^^73^ Of practical significance, use of Eq. (7) on our AO-OCT data underestimates the true speckle contrast, a consequence of dewarping the B-scans in postprocessing to correct for nonlinearity of the scan pattern.

## Appendix C: Effectiveness of Our Method to Remove Structural Correlation Bias from Time Constant Measurements

**Fig. 11:**
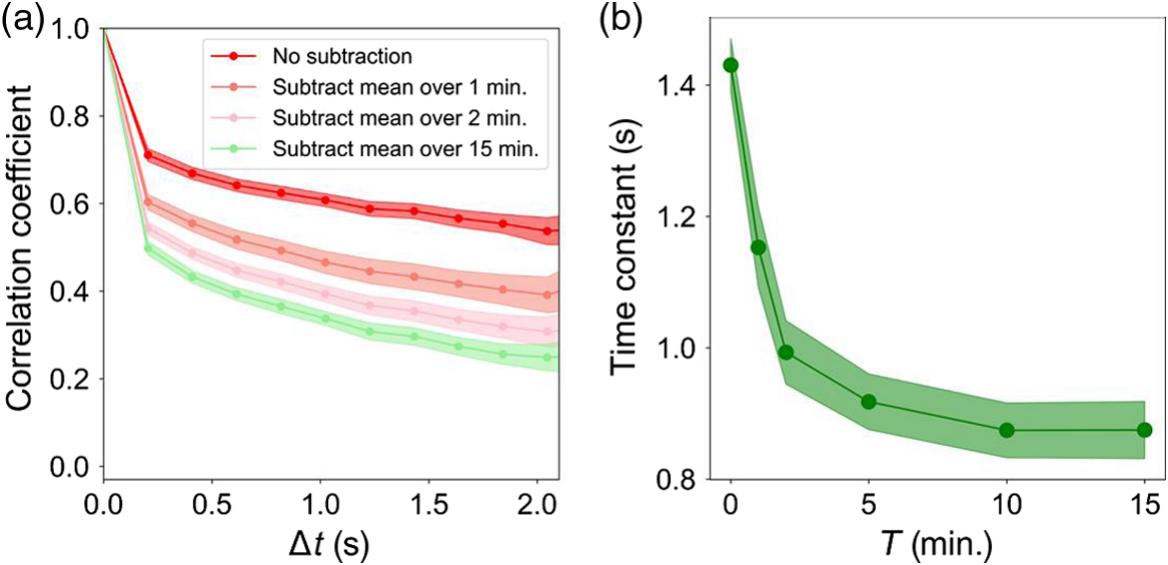
Effectiveness to remove structural correlation from the NFL correlation coefficient and time constant improves with increased averaging time period, T. (a) Full-layer (window #1) temporal correlation coefficient $\rho (\overset{\to }{r_{c}},\Delta t)$ and (b) corresponding time constant for T=0, 1, 2, 5, 10, and 15 min. Shaded colored bands about each trace denote 95% confidence intervals.

We evaluated the effectiveness of our method [see Appendix A, Eq. (2)] to remove structural correlation bias (time-invariant contributions) from our time constant measurements. To determine, we computed the temporal correlation coefficient of NFL using window #1 (300×300×7  pixel stack) on the same dataset shown in Fig. 1(f) (subject S1), whereas varying the averaging time period T used in Appendix A, Eq. (2). Figure 11 shows the resulting temporal correlation coefficient and corresponding time constant for T=0, 1, 2, 5, 10, and 15 min. As evident in the plots, short averaging periods (T<5  min) overestimate the time constant, but this error decreases asymptotically and becomes negligible for averaging periods T>10  min. We obtained similar results for GCL and IPL. Thus, we conclude that the T=15  min averaging period used in our study is sufficient to remove structural biases in the layers, tissues, and cells we examined.

## Appendix D: Confirm Correlation Coefficient Reaches Zero for Long Time Periods

**Fig. 12:**
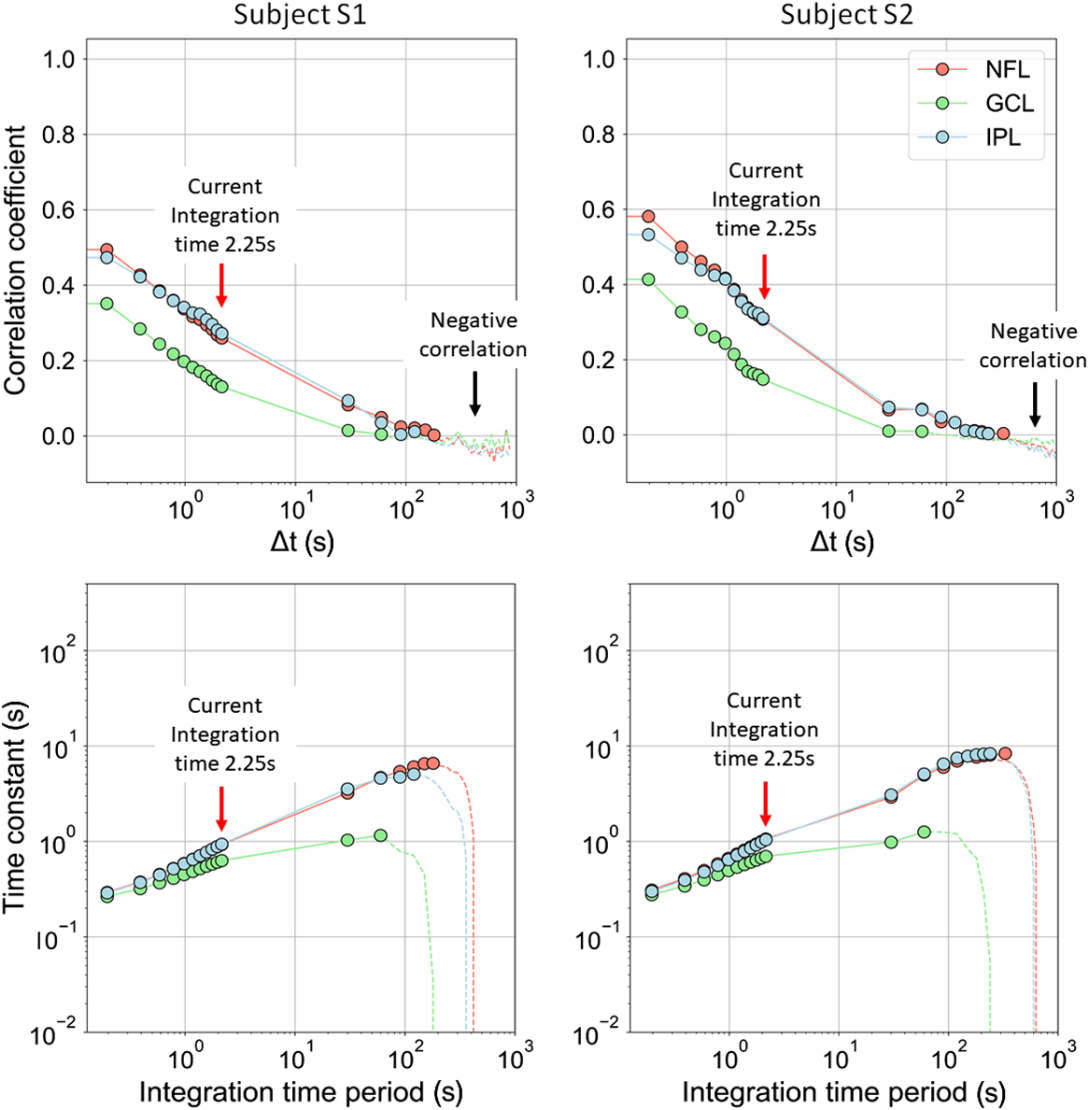
(top row) Temporal correlation coefficients, $\rho (\overset{\to }{r_{c}},\Delta t)$, and (bottom row) corresponding time constants of NFL, GCL, and IPL data in Fig. 1 are shown over an extended time period of $∼10^{3}\;s$. Window #1 was used. Note that the gap between data points 2.25 and 30 s is the time interval between consecutively acquired AO-OCT videos in which no data were acquired. The red arrows indicate the integration time period used in this study. The black arrows and dashed lines indicate where negative correlation occurs. A negative correlation indicates an unreliable estimate of the time constant and should be ignored.^33^

The correlation coefficients determined in Figs. 1(f) and 1(l) did not reach zero for the 2.25-s interval shown. Since in theory the coefficients must for a long enough time period, we tested this limit by recomputing the correlation coefficients after incorporating additional AO-OCT volume images that extended the time interval to 1000 s, almost 500× longer than the 2.25 s used in our study. To extend, we concatenated videos that were acquired consecutively of the same retinal patch and recorded 30 to 240 s apart (see Table 2). Figure 12 (top row) shows our extended correlation results for the inner retinal layers (NFL, GCL, and IPL). As evident for both subjects, the layers decorrelate to less than 0.1 after ∼30  s and reach zero after about 60 to 330 s depending on the layer. Thus, we confirm our method can detect zero correlation.

Next we computed time constants from the Fig. 12 (top row) correlation coefficient traces using Eq. (5) in Appendix A and plotted them in Fig. 12 (bottom row) on a log-log scale. As shown, the time constant strongly depends on the integration time period, varying from 0.3 s with the shortest integration period (T=0.3  s) to 8.4 s with the longest (T=330  s). This monotonic increase attributes from the shallow, nonexponential decay trace of our correlation coefficients that exhibit appreciable energy at low temporal frequencies (periods > 5  s). As such increasingly longer integration periods capture increasingly lower temporal frequencies that increase the time constant. As this dependence is fundamental to the correlation theory used, a common approach to circumvent it is to select a fixed integration period for all measurement comparisons. For this study, we selected T=2.25  s as this captures the period of most rapid change in the correlation coefficients of NFL, GCL, and IPL.

## Appendix E: Determine Decorrelation Bias of Residual (subpixel) Eye Motion for Correcting Time Constant Measurements

Our image registration algorithm corrects motion artifacts as small as a single image pixel. We therefore expect $\varepsilon_{i}^{2}$ (residual displacement error) in Eq. (6) of Appendix A to be limited by the sample spacing, i.e., the 1-μm/pixel spacing used in our study. This limitation has been demonstrated in previous studies under the assumption of fully developed speckle.^74^^–^^76^ With this assumption, we estimated the size of $\varepsilon_{i}^{2}$ by comparing correlation differences measured for two different sample spacings: 1 and 1.5  μm/pixel. This approach follows that used in speckle metrology to measure surface roughness by purposefully changing speckle size, wavelength, or illumination angle by a known amount.^26^ To evaluate, we measured the same retinal patches of the same subjects using imaging protocols A and C (Table 1). Protocol C gave the same SNR ratio and A-scan exposure duration as protocol A but with coarser spatial sampling (1.5  μm/A-scan instead of 1  μm/A-scan). Because image registration accuracy is limited to pixel size, the coarser sampling should increase the residual displacement error (measured in microns, not pixels).

**Fig. 13:**
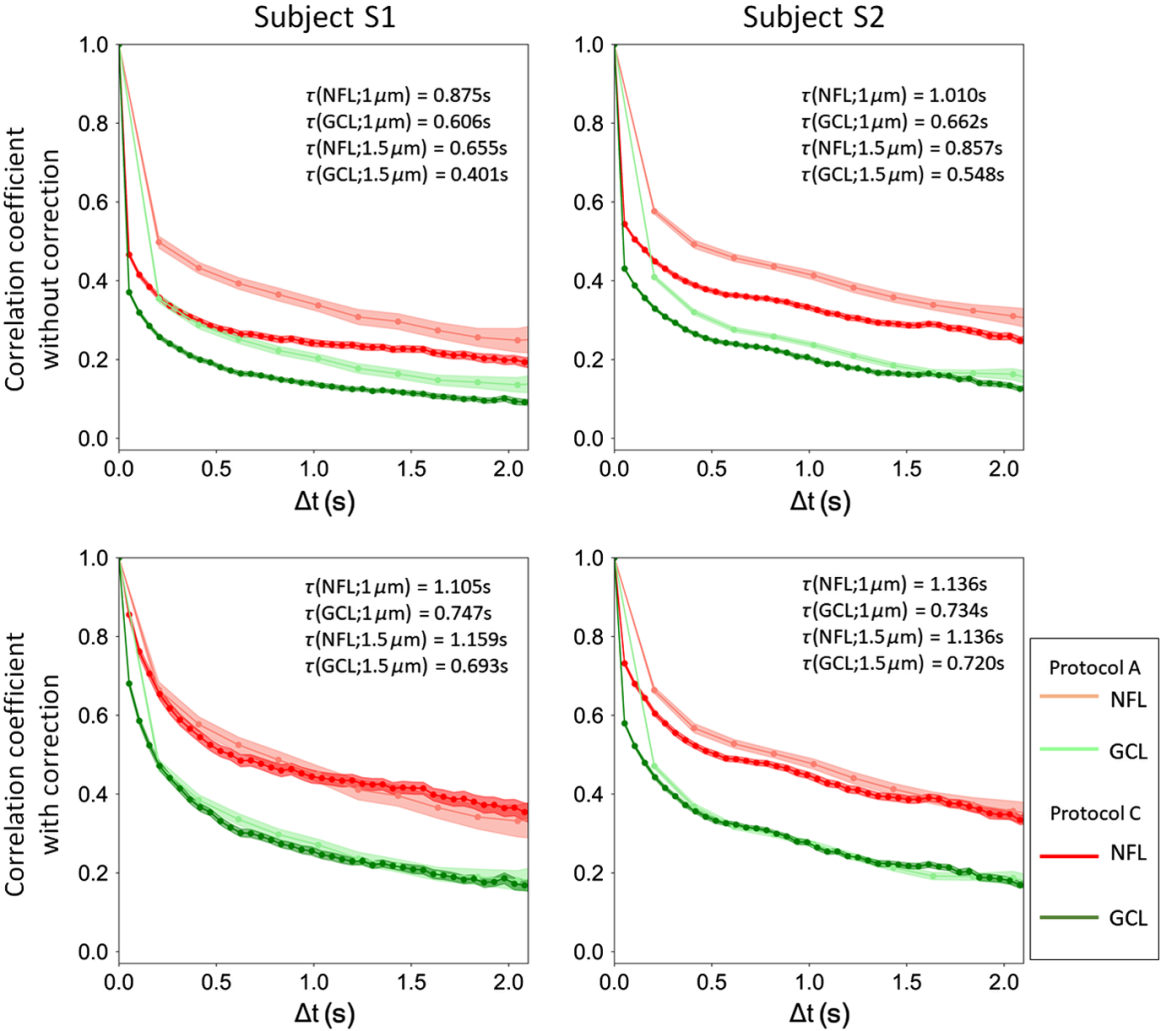
Temporal correlation coefficients $\rho (\overset{\to }{r_{c}},\Delta t)$ before (top row) and after (bottom row) correction of residual eye motion bias. Correlation coefficients are shown for two sample spacings (1 and 1.5  μm/A-scan) for two of the inner retinal layers (NFL and GCL) of both subjects, S1 and S2. Shaded colored bands about each trace denote 95% confidence intervals. See text for details.

Figure 13 (top row) shows the resulting temporal correlation coefficients of the inner retinal layers (NFL and GCL) computed using the two protocols with window #1 (300×300×7  pixel stack and 150×150×7  pixel stack, respectively). Time constants are listed in each plot. The coarser sampled measurements have time constants that are 27% and 14% lower in subjects S1 and S2, respectively. Assuming this reduction is due entirely to residual displacement errors as described by Eq. (6) of Appendix A, we solved for $\varepsilon_{i}^{2}$, resulting in a $\varepsilon_{i}$ of 0.86  μm (subject S1) and 0.60  μm (subject S2) for 1-μm/pixel sampling. For both subjects, $\varepsilon_{i}$ is near the 1-μm/pixel sampling, indicating our image registration algorithm corrected eye motion at the pixel resolution of our method, as we had expected. Next, we estimated $\sigma_{\rho }^{2}$ for both subjects using the $\varepsilon_{i}$ values with Eq. (6) and then corrected this bias in our original correlation coefficients traces [see Fig. 13 (bottom row), with and without the $\sigma_{\rho }^{2}$ correction]. As evident in the plots, correction of residual displacement errors results in shallower decaying correlation coefficients and increased time constants that are insensitive to sample spacing (1 and 1.5  μm/pixel).

## Appendix F: Estimate Random Motion of Intracellular Scatterers from Time Constant Measurements

Temporal correlation measurements are sensitive to fluctuations in light scattered from moving particles (e.g., intracellular organelles) that occupy the coherence volume of the AO-OCT beam ($2.4\times 2.4\times 4.7\;\mu m^{3}$). Following Berne and Pecora,^33^ we modeled this diffusive motion as a simple random walk and from which the correlation decay can be described by an exponential: $\exp[-\Delta t\cdot \underset{i=x,y,z}{\sum }{\left(\sigma_{i}/w_{i}\right)}^{2}]$, where Δt is the temporal sampling interval of our AO-OCT (0.19  s/volume), $w_{i}$ is the speckle size of our AO-OCT system [2.4, 2.4, and 4.7  μm for i=x,y,z in retinal tissue, and $\sigma_{i}^{2}$ is the random motion variance of scatterers in the retinal tissue that occurs per second in each direction (i=x,y,z)]. We relate this correlation decay expression to the time constant by $\exp(-\Delta t/\tau )∼\exp[-\Delta t\cdot \underset{i=x,y,z}{\sum }{\left(\sigma_{i}/w_{i}\right)}^{2}]$ and solve for $\sigma_{i}^{2}$ to obtain an estimate for the random motion in each direction as described by $\sigma_{i}^{2}∼\underset{i=x,y,z}{\sum }w_{i}^{2}/3\tau$ ($\mu m^{2}/s$). From this expression and our AO-OCT’s measured range of τ (0.38 to 2.25 s), we determined the range of random scatterer motion that we could measure: 5 to $29\;\mu m^{2}/s$ [or 2.5 to $15\;\mu m^{2}/s$ for the equivalent diffusion coefficient ($\sigma_{i}^{2}/2$)].

## Appendix G: Repeated Measures of the Correlation Coefficients

In Secs. 3.1 and 4.1.1, significant differences were found between retinal layers, sublayer tissues, and individual cells. For example, the two-way ANOVA test for variations in τ with retinal layer and subject showed that residual sum of squares (equivalent to repeatability errors) $\sigma_{e}^{2}$ was just 10% of total sum of squares ($\sigma_{\tau }^{2}$). Thus, a small fraction (10%) of the total variance is attributed to repeatability error.

**Fig. 14:**
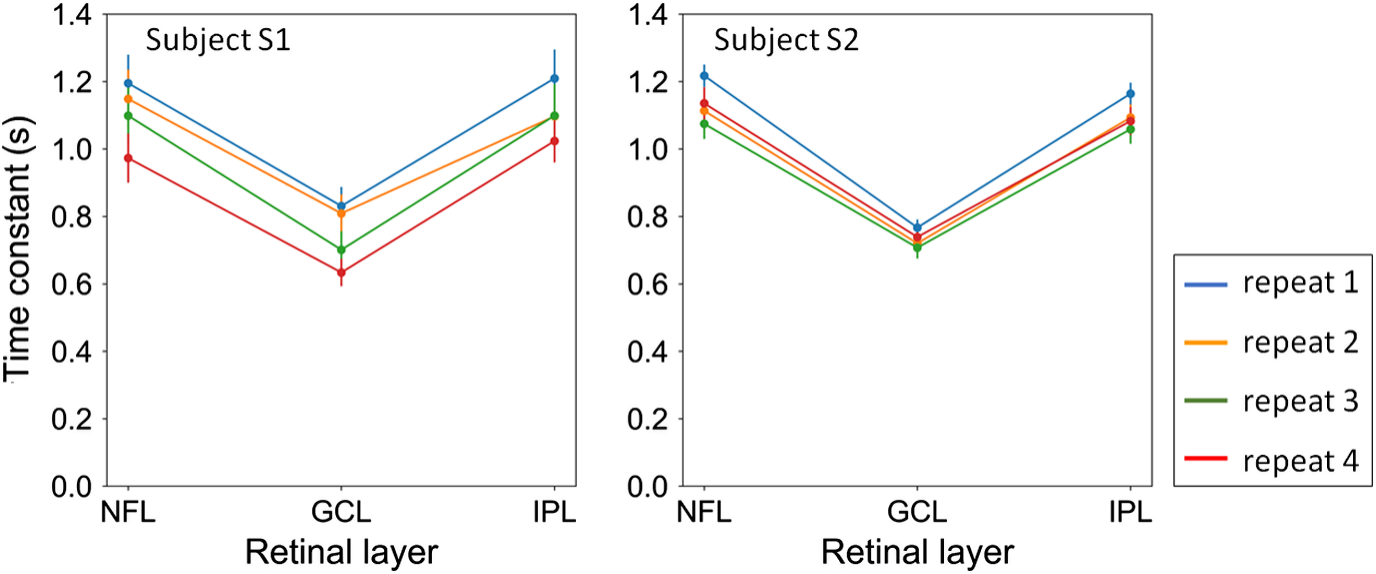
Four repeated measures of time constant across the three retinal layers (NFL, GCL, and IPL) and two subjects (S1 and S2). Error bars denote the standard deviation for each repeat.

To further substantiate this finding, we repeated the study by measuring the same retinal patches of the same subjects at four different time points using imaging protocol A (see Table 2) Each time point consisted of five to six videos acquired within 6 min. Results were analyzed for variations in τ with retinal layer and subject at each time. We tracked fast temporal dynamics up to 2.6 Hz (Nyquist frequency of the 5.3-Hz volume acquisition rate) over a 1-deg retinal field of view and with 1  μm/A-scan lateral spacing. For simplicity, we computed the temporal correlation coefficients of the entire layers using window #1. Figure 14 shows the time constants measured at the four time points. Despite fewer numbers of independent samples than in the main study (Sec. 3.1), we found the main effect of retinal layers to be significant for all the time points (p<0.001). The main effect of subjects and the interaction between subjects and layers were significant in two of the four time points but not for the same points. Bonferroni-adjusted comparisons indicated that the time constant of GCL was faster than that of NFL and IPL of both subjects at all time points (p<0.001). Thus, we conclude that differences between retinal layers are significant and repeatable. Differences between subjects are too small for our repeatability test, as designed, to measure.
